# Hepatic lipid profile in mice fed a choline-deficient, low-methionine diet resembles human non-alcoholic fatty liver disease

**DOI:** 10.1186/s12944-020-01425-1

**Published:** 2020-12-09

**Authors:** Elisabeth M. Haberl, Rebekka Pohl, Lisa Rein-Fischboeck, Marcus Höring, Sabrina Krautbauer, Gerhard Liebisch, Christa Buechler

**Affiliations:** 1 Department of Internal Medicine I, Regensburg University Hospital, Regensburg, Germany; 2 Institute of Clinical Chemistry and Laboratory Medicine, Regensburg University Hospital, Regensburg, Germany

**Keywords:** Liver tumor, Ceramide, Phospholipids, Lipogenesis, Cholesterol, Diethylnitrosamine

## Abstract

**Background:**

Emerging data support a role for lipids in non-alcoholic steatohepatitis (NASH) and hepatocellular carcinoma (HCC) in humans. With experimental models such data can be challenged or validated. Mice fed a low-methionine, choline-deficient (LMCD) diet develop NASH and, when injected with diethylnitrosamine (DEN), HCC. Here, lipidomic analysis was used to elucidate whether the NASH and HCC associated lipid derangements resemble the lipid profile of the human disease.

**Methods:**

Lipids were measured in the liver of mice fed a control or a LMCD diet for 16 weeks. DEN was injected at young age to initiate hepatocarcinogenesis. DEN treatment associated changes of the lipid composition and the tumor lipidome were evaluated.

**Results:**

LMCD diet fed mice accumulated ceramides and triacylglycerols in the liver. Phospholipids enriched with monounsaturated fatty acids were also increased, whereas hepatic cholesterol levels remained unchanged in the LMCD model. Phosphatidylcholine and lysophosphatidylcholine concentrations declined in the liver of LMCD diet fed mice. The changes of most lipids associated with LMCD diet feeding were similar between water and DEN injected mice. Several polyunsaturated (PU) diacylglycerol species were already low in the liver of DEN injected mice fed the control diet. Tumors developed in the liver of LMCD diet fed mice injected with DEN. The tumor specific lipid profile, however, did not resemble the decrease of ceramides and PU phospholipids, which was consistently described in human HCC. Triacylglycerols declined in the cancer tissues, which is in accordance with a low expression of lipogenic enzymes in the tumors.

**Conclusions:**

The LMCD model is suitable to study NASH associated lipid reprogramming. Hepatic lipid profile was modestly modified in the DEN injected mice suggesting a function of these derangements in carcinogenesis. Lipid composition of liver tumors did not resemble the human HCC lipidome, and most notably, lipogenesis and triacylglycerol levels were suppressed.

**Supplementary Information:**

The online version contains supplementary material available at 10.1186/s12944-020-01425-1.

## Introduction

Non-alcoholic steatohepatitis (NASH) is a considerable risk factor for liver fibrosis and hepatocellular carcinoma (HCC) [1–3]. Dietary animal models are widely used to study NASH pathogenesis and feeding mice a chow deficient in choline and methionine is the method of choice [4]. Unfortunately, by two weeks animals loose about 10 to 20% of their body weight [4–6]. To overcome excessive weight loss, the L-amino acid-defined, choline-deficient, dietary mouse model was established [7, 8]. These animals maintain body weight and develop NASH after 8 to 12 weeks. NASH promotes liver carcinogenesis and injection of the carcinogen diethylnitrosamine (DEN) combined with a L-amino acid-defined, choline-deficient diet makes it a useful NASH-HCC model [7–9].

Dysregulated lipid metabolism is a hallmark of NASH [10]. Derivatives of fatty acids and non-triglyceride lipids cause lipotoxic injury [10]. Ceramides, diacylglycerols and lysophosphatidylcholines can induce endoplasmatic reticulum stress, inflammation and cell death [10].

The biologic function of lipids is determined by the acyl-chain length. Whereas long-chain ceramides (C16 - C20) induced insulin resistance, apoptosis and oxidative stress, the very long-chain derivatives (C22 - C24) had the opposite effects [11, 12]. Thus detailed analysis of ideally all lipid derivatives is essential for understanding lipotoxicity in liver diseases [10].

Emerging evidence suggests that cholesterol is a risk factor for NASH and accumulation of free cholesterol activates inflammatory and fibrotic pathways in the liver [13]. The ratio of phosphatidylcholine to phosphatidylethanolamine is a valid marker of plasma membrane integrity and decreased along with the progression of NASH [14].

NASH is a risk factor for HCC, where an epithelial to mesenchymal transition (EMT) process occurs during the progression of HCC [11, 15]. Lipid metabolism reprogramming in mesenchymal cells is under the control of β-catenin and these cells mostly use exogenous fatty acids for triacylglycerol synthesis by diacylglycerol acyltransferase 1 (DGAT1) [15]. Accumulation of saturated triacylglycerols and phospholipids, and depletion of ceramides and polyunsaturated fats in HCC tissues protects the tumor cells from apoptosis and oxidative stress [11].

Animal experiments are still needed and choosing the appropriate preclinical model is still a challenge [16]. NASH and HCC are heterogeneous diseases, and mouse models can reflect only some aspects of the human disease [4, 7, 17].

Owing to the significant role of lipids in NASH and cancer [10, 11], aim of the present study was a comprehensive lipidomic analysis of non-tumorous and tumor tissues of mice injected with DEN and fed a low-methionine, choline-deficient (LMCD) diet. Therefore, liver tissues of recently described mice were used [9]. The LMCD diet fed mice had hepatic steatosis and fibrosis. Body weight and subcutaneous fat mass were not changed by LMCD diet feeding whereas epididymal fat mass was increased [9]. The distribution of body fat plays an eminent role in NAFLD pathogenesis and growth of intra-abdominal adipose tissue contributes to liver injury [2]. DEN injection induced carcinogenesis, with much more liver tumors developing in the LMCD diet fed group [9].

## Materials and methods

### Animals

Male C3H/HeNRj mice were from Janvier Labs (Le Genest-Saint-Isle, France). The 18- to 21-day-old animals were injected intraperitoneally with 25 μg DEN/g body weight (Sigma, Taufkirchen, Germany), which was dissolved in water. Injection of water served as a control. After feeding a standard chow for 5 weeks, animals were kept on a control chow (E15668–04; Ssniff, Soest, Germany) or a low-methionine, choline-deficient (LMCD) diet (E15667–94; Ssniff) for 16 weeks. Animals had free access to water and food. Mice were housed at 21 ± 1 °C under a 12 h light-dark cycle. At the age of 24 weeks mice were killed under CO_2_-induced coma by cervical dislocation. The livers of 11 water-injected animals fed a control diet, of 11 water-injected mice fed the LMCD diet, of 10 DEN-injected animals fed the control diet and 11 mice with DEN administration and LMCD chow were used for lipid analysis.

Macroscopically visible tumors were excised from the livers using a pair of binoculars. Most of the tumors had a diameter of less than 0.4 mm [9]. Diameter of five tumors was larger (2.5, 3.2, 3.4, 4.0 and 4.0 mm) and the corresponding tumor wet weight was 20, 28, 33, 36 and 50 mg, and these tissues were used for lipid analysis.

These mice were described in detail in a recent study where the expression of chemerin was analysed [9].

### Mass spectrometric analysis

The analysis of lipids was performed by direct flow injection analysis (FIA) using a triple quadrupole mass spectrometer (FIA-MS/MS; QQQ triple quadrupole) and a hybrid quadrupole-Orbitrap mass spectrometer (FIA-FTMS; high mass resolution).

FIA-MS/MS was performed in positive ion mode using the analytical setup and strategy described previously [18, 19]. A fragment ion of *m/z* 184 was used for lysophosphatidylcholines (LPC) [20]. The following neutral losses were applied: phosphatidylethanolamine (PE) 141, phosphatidylserine (PS) 185, phosphatidylglycerol (PG) 189 and phosphatidylinositol (PI) 277 [21]. PE-based plasmalogens (PE P) were analyzed according to the principles described by Zemski and Murphy [22]. Sphingosine based ceramides (Cer) were analyzed using a fragment ion of *m/z* 264 [23].

The Fourier Transform Mass Spectrometry (FIA-FTMS) setup is described in Höring et al. [24]. Triacylglycerol (TG), diacylglycerol (DG) and cholesteryl ester (CE) were recorded in positive ion mode FTMS in range *m/z* 500–1000 for 1 min with a maximum injection time (IT) of 200 ms, an automated gain control (AGC) of 1*10^6^, three microscans and a target resolution of 140,000 (at *m/z* 200). Phosphatidylcholine (PC) and sphingomyelin (SM) were measured in range *m/z* 520–960. Multiplexed acquisition (MSX) was used for the [M + NH_4_]^+^ of free cholesterol (FC) (*m/z* 404.39) and D_7_-cholesterol (*m/z* 411.43) for 0.5 min acquisition time, with a normalized collision energy of 10%, an IT of 100 ms, AGC of 1*10^5^, isolation window of 1 Da, and a target resolution of 140,000. Data processing using the ALEX software [25] and self-programmed Macros (Microsoft Excel 2010) was described previously [26]. Lipid species were annotated according to the proposal for shorthand notation of lipid structures that are derived from mass spectrometry [27]. For QQQ glycerophospholipid species annotation was based on the assumption of even numbered carbon chains only. Liver lipids are denoted as nmol/mg wet weight.

### Lipid extraction

Lipid extraction was performed according to the method of Bligh and Dyer [28] in the presence of non-naturally occurring lipid species as internal standards. The following lipid species were added as internal standards: PC 14:0/14:0, PC 22:0/22:0, PE 14:0/14:0, PE 20:0/20:0 (di-phytanoyl), PS 14:0/14:0, PS 20:0/20:0 (di-phytanoyl), PI 17:0/17:0, LPC 13:0, LPC 19:0, LPE 13:0, Cer d18:1/14:0, Cer d18:1/17:0, D7-FC, CE 17:0, CE 22:0, TG 51:0, TG 57:0, DG 28:0 and DG 40:0. Liver homogenates containing a wet weight of 2 mg were used for lipid extraction. The chloroform phase was recovered, vacuum dried and the contents were dissolved either in methanol/chloroform (3:1, v/v) with 7.5 mM ammonium acetate (for low mass resolution tandem mass spectrometry) or methanol/chloroform/2-propanol (1:2:4 v/v/v) with 7.5 mM ammonium formate (for high resolution mass spectrometry).

### Immunoblotting, ELISA and analysis of malondialdehyde levels

Immunoblotting was carried out as described [9]. Antibodies for beta-actin, glyceraldehyde-3-phosphate dehydrogenase (GAPDH), fatty acid synthase (FAS), alpha-smooth muscle actin (alpha-SMA), collagen 1A1 (Col1A1) and stearoyl-CoA desaturase 1 (SCD1) were from New England Biolabs GmbH (Frankfurt am Main, Germany). Manganese superoxide dismutase (MnSOD) antibody was from Thermo Fisher Scientific (Schwerte, Germany). Low density lipoprotein (LDL)-receptor antibody, diacylglycerol-O-acyltransferase (DGAT) 1 antibody and acetyl-CoA-carboxylase (ACC) antibody were from Novus Biologicals (Centennial, CO, USA). The 4-hydroxynonenal (4-HNE) and p53 antibodies were from R&D Systems (Wiesbaden-Nordenstadt, Germany). Immunoblots were quantified with Image J [29]. ELISA to measure alpha-fetoprotein (AFP) was from R&D Systems and serum was diluted 20-fold. Malondialdehyde levels were determined by a colorimetric assay from Abcam (Lipid Peroxidation (MDA) Assay Kit).

### Statistical analysis

Individual data points and the median values are shown in the graphs. Statistical differences were calculated by one-way ANOVA with post-hoc Bonferroni or Mann-Whitney U-test (SPSS Statistics 25.0 program; IBM, Leibniz Rechenzentrum, Munich, Germany) and differences with values of *P* <  0.05 were considered as significant.

## Results

### Mice fed the LMCD diet develop liver fibrosis and have high serum alpha-fetoprotein when injected with DEN

**Fig. 1:**
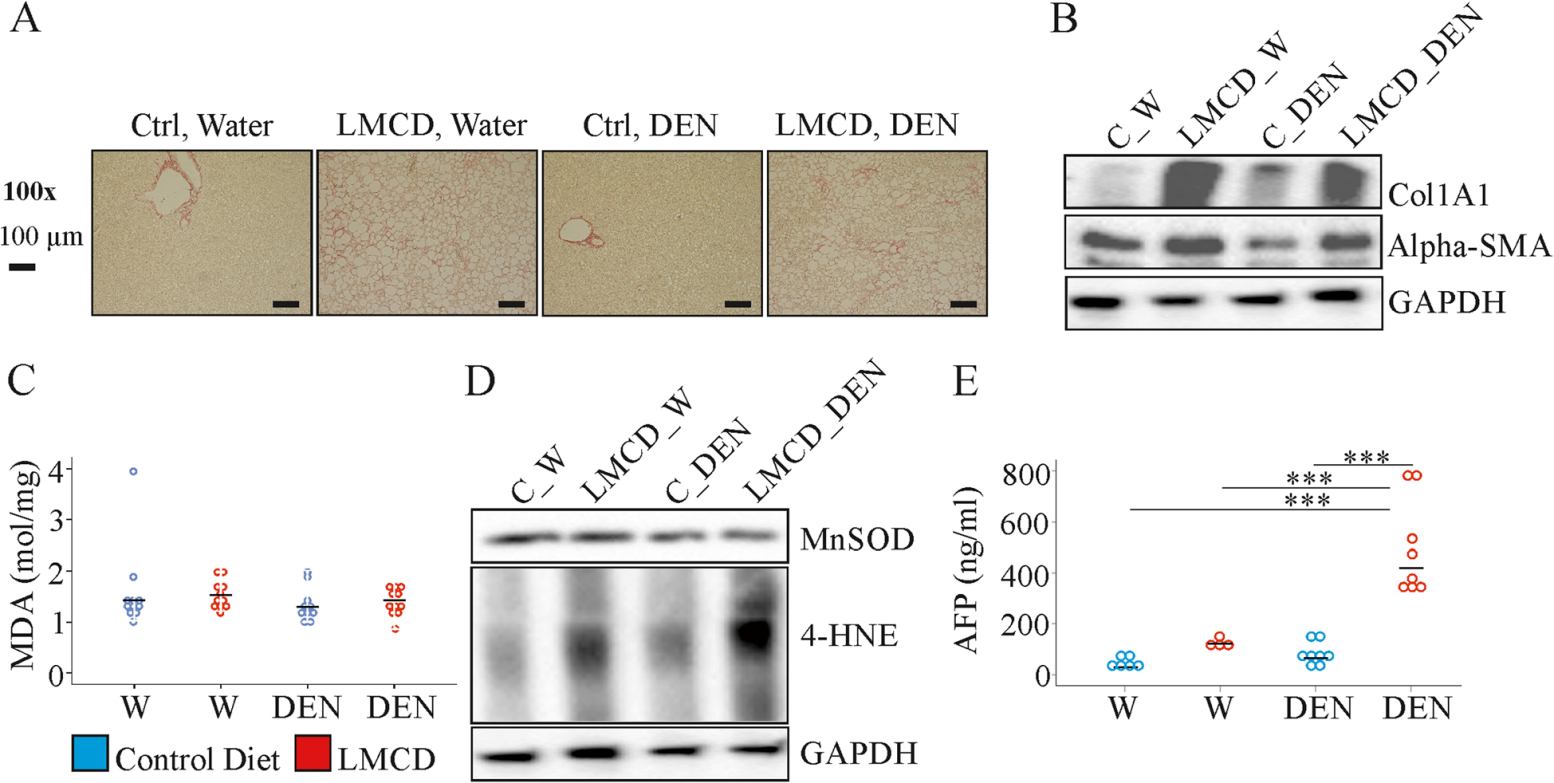
Fibrosis and oxidative stress markers in the liver and serum alpha-fetoprotein (AFP) of mice fed a control or low-methionine choline-deficient (LMCD) diet, and injected either with water (W) or diethylnitrosamine (DEN) (*n* = 4–8 mice per group). **a** Sirius red stained liver of the animals, **b** Collagen 1A1 (Col1A1) and alpha-smooth muscle actin (alpha-SMA) protein in the liver, **c** hepatic MDA levels, **d** MnSOD and 4-HNE in the liver, **e** AFP measured in serum. *** *P* <  0.001

This study aimed at a comprehensive analysis of the liver lipidome of mice fed a LMCD diet to identify similarities to the lipid profiles of human NASH. Therefore, liver tissues of mice injected with water as control or DEN and fed the appropriate control chow or the LMCD diet were used [9]. All of the mice on the LMCD diet had liver fibrosis. This was evaluated by Sirius Red staining [9] and analysis of collagen 1A1 and alpha-SMA protein in the liver (Fig. 1a, b). MDA and 4-HNE are lipid peroxidation products, and the latter was increased in the liver of LMCD diet fed mice (Fig. 1c, d and Supplementary Table 1). The antioxidant enzyme MnSOD was neither changed by diet nor DEN injection (Fig. 1d and Supplementary Table 1). Liver cancers developed in the DEN treated mice and LMCD diet was associated with a five-fold higher tumor number [9]. Alpha-fetoprotein (AFP) is a biomarker for HCC [30] and was increased in serum of DEN injected mice fed the LMCD diet (Fig. 1e). These data show that DEN injection induced carcinogenesis but did not worsen oxidative stress and liver fibrosis in the NASH model.

### Mice fed the LMCD diet accumulate triacylglycerols in the liver even though acetyl-CoA-carboxylase is downregulated

**Fig. 2:**
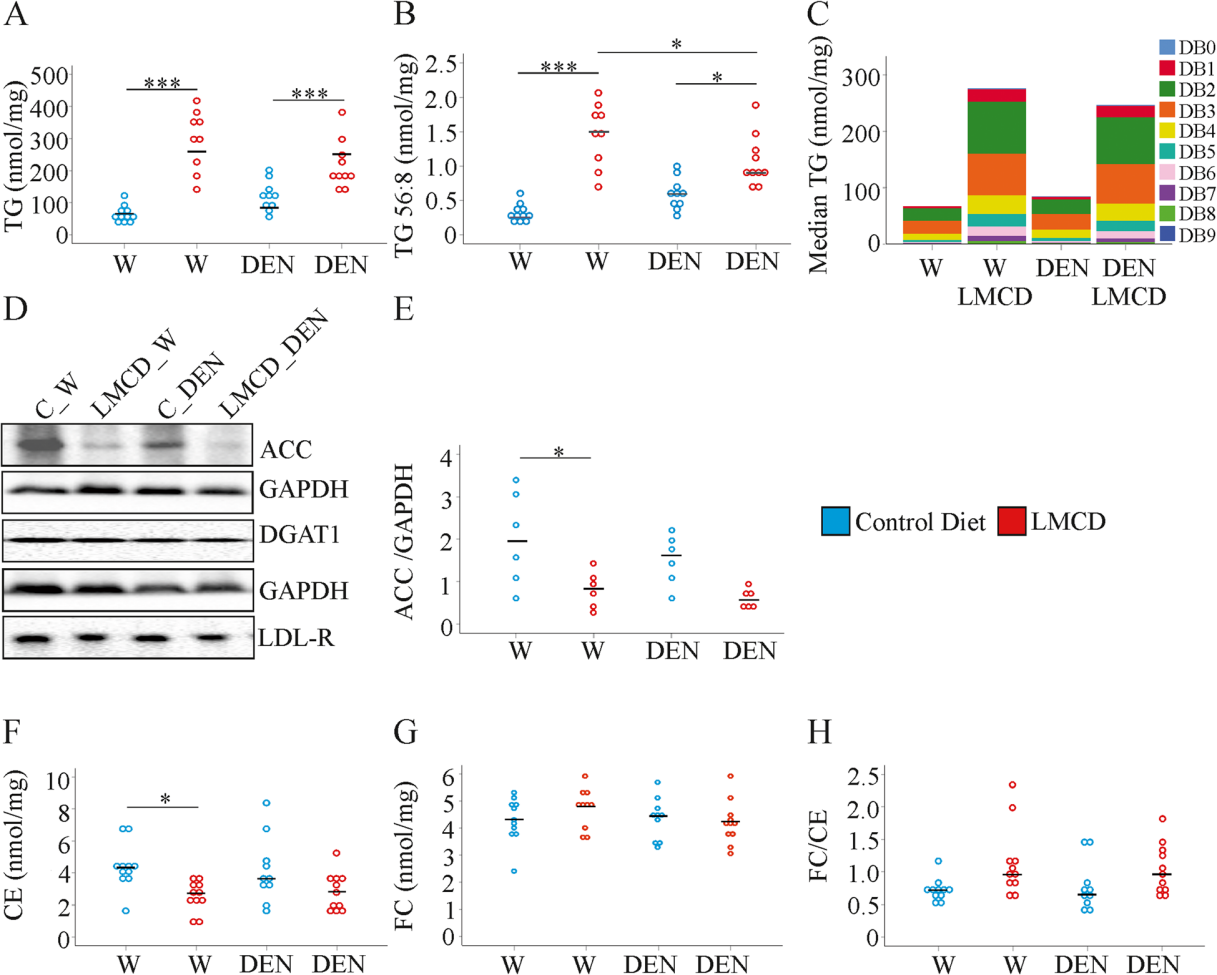
Levels of triacylglycerol (TG), cholesterol, and expression of proteins that are involved in lipid metabolism, in the liver of mice fed a control or low-methionine choline-deficient (LMCD) diet, and injected either with water (W) or diethylnitrosamine (DEN) (*n* = 8–11 mice per group). **a** TG, **b** TG 56:8, **c** Median values of TGs with 0 to 9 double bonds in the liver of the mice, **d** Expression of ACC, DGAT1 and the LDL-R in the liver, **e** Quantification of ACC protein in the liver, **f** Cholesteryl ester (CE), **g** Free cholesterol (FC), **h** FC / CE ratio. * *P* <  0.05, *** *P* <  0.001

Hepatic triacylglycerol (TG) accumulation is a characteristic of liver steatosis, and was observed in the LMCD model (Fig. 2a). All of the measured TG species were increased in the liver of LMCD diet fed mice irrespective of DEN injection (Supplementary Table 2). This upregulation was significant for all TG species except TG 48:0 in the liver of the DEN-treated animals (Supplementary Table 2). In the LMCD models, hepatic TG 54:6 and 56:8 were lower in the DEN-injected mice (Fig. 1b and Supplementary Table 2).

TGs with 0, 1, 2, 3, 4, 5, 6, 7, 8 and 9 double bonds were increased in the NAFLD liver of water and DEN injected mice (Fig. 1c). Distribution of the TGs with varying degrees of unsaturation did not differ between the water and DEN injected groups (Fig. 1c).

There were small variations in the proportion of several TG species in the total TG fraction of the control diet fed mice. Here, four TG species (TG49:1, 50:1, 50:2, 50:3) were lower in the liver of the DEN treated mice and two species (TG 56:8, TG 58:8) were induced (Supplementary Table 3). In the LMCD diet fed mice the percentage of all TG species (relative to total TG levels) was similar between the two groups (data not shown).

Acetyl-CoA-carboxylase (ACC) catalyses the synthesis of malonyl CoA from acetate [31]. ACC protein was reduced in the liver of LMCD fed mice, and this effect was significant in the water-injected group (Fig. 2d, e and Supplementary Table 1). ACC and fatty acid synthase (FAS) are both regulated by sterol regulatory element-binding protein-1c [31] and FAS protein was markedly reduced in the liver of LMCD fed mice [9]. Diacylglycerol-O-acyltransferase 1 (DGAT1) catalyzes the last step in the synthesis of TGs and high expression resulted in increased hepatic triglyceride levels [32]. Immunoblot analysis revealed that the DGAT1 protein was not altered in the liver of mice fed the LMCD diet (Fig. 2d and Supplementary Table 1). Overall, these data suggest that hepatic lipogenesis was impaired in the LMCD model. Esterification of exogenous fatty acids by DGAT1 seems to contribute to hepatic TG accumulation.

Sterol regulatory element-binding protein 2 (SREBP2) controls cholesterol synthesis and was downregulated in the liver of LMCD diet fed mice. SREBPs also regulate the LDL receptor, which mediates uptake of systemic cholesterol into the liver [9, 31]. LDL-receptor protein was, however, not reduced in the liver of the LMCD fed mice (Fig. 2d and Supplementary Table 1).

Cholesteryl ester levels declined in the liver of the LMCD diet fed mice and this was significant in the water-injected mice (Fig. 2f and Supplementary Table 4). Total hepatic cholesterol levels, free cholesterol (FC) concentration and ratio of FC/cholesteryl ester were neither changed by diet nor by DEN treatment (Fig. 2g, h and Supplementary Table 4).

### Mice fed the LMCD diet have increased hepatic ceramide levels

**Fig. 3:**
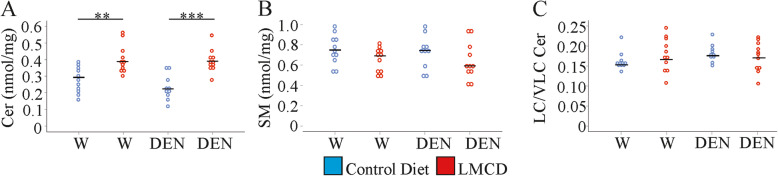
Hepatic ceramide (Cer) and sphingomyelin (SM) levels in mice fed a control or low-methionine choline-deficient (LMCD) diet, and injected either with water (W) or diethylnitrosamine (DEN) (*n* = 10–11 mice per group). **a** Cer, **b** SM, **c** long-chain (LC) / very long-chain (VLC) Cer ratio. ** *P* <  0.01, *** *P* <  0.001

Ceramides regulate insulin signalling and apoptosis, and were induced in human NASH liver [33]. Total ceramide levels were also increased in the liver of the LMCD diet fed mice irrespective of DEN injection (Fig. 3a). Sphingomyelinases remove the phosphocholine group from sphingomyelin (SM) to produce ceramides [34]. Since higher ceramides were not accompanied by reduced SM levels in the liver of LMCD diet fed mice, this pathway was not involved herein (Fig. 3b). Higher ceramides in the murine NASH liver thus originated from de novo synthesis, which is in line with findings in NAFLD patients [35].

Ceramide acyl-chain length greatly alters the biologic function of these lipids [11, 12]. Ratio of long-chain to very long chain ceramide species was neither changed by diet nor by DEN treatment (Fig. 3c). The equal change of these different ceramide species does not exclude a pathophysiological role of ceramide accumulation in the liver.

### Mice fed the LMCD diet have a reduced hepatic phosphatidylcholine / phosphatidylethanolamine ratio and increased levels of monounsaturated phospholipids

**Fig. 4:**
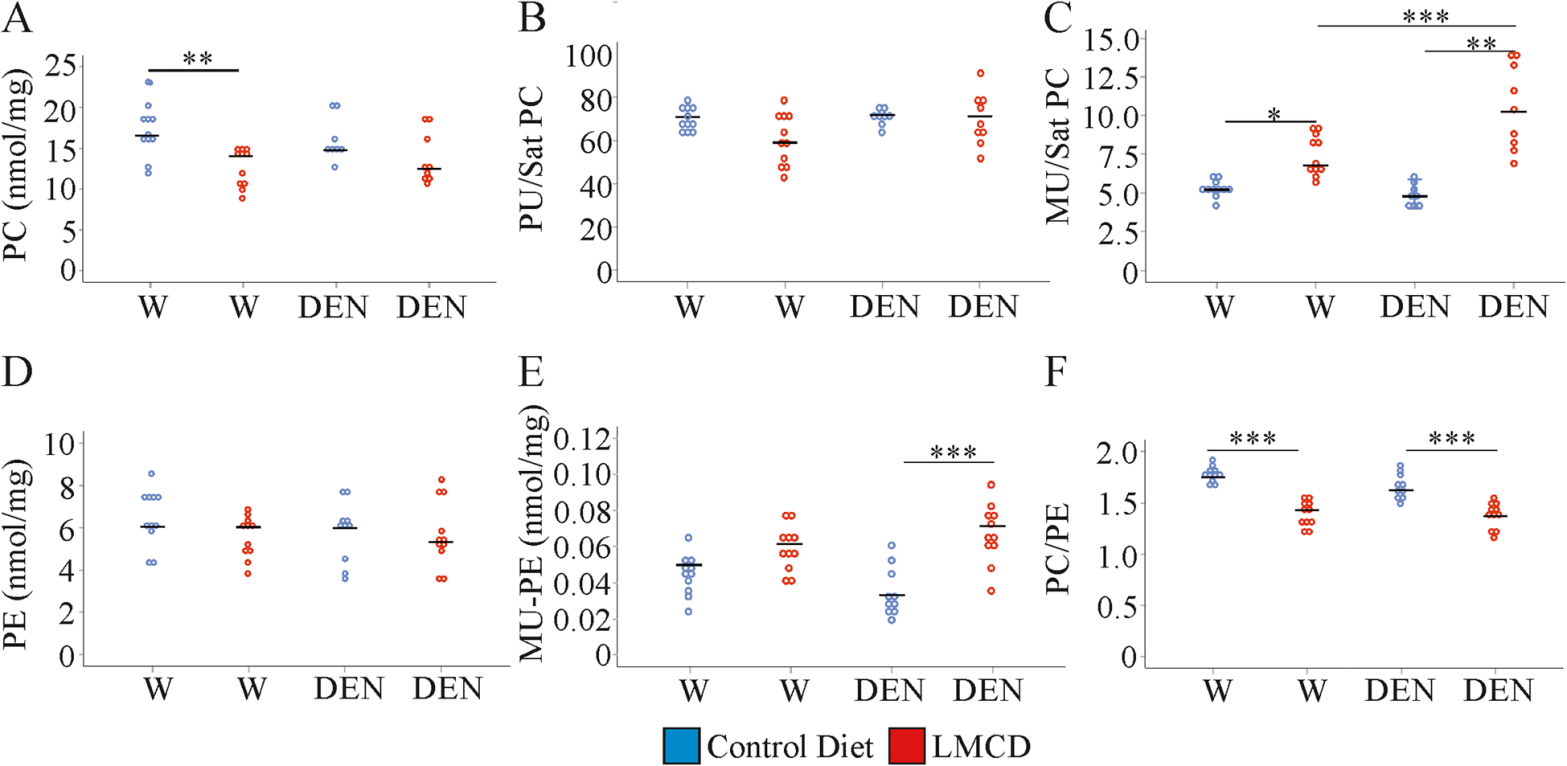
Hepatic phosphatidylcholine (PC) and phosphatidylethanolamine (PE) levels in mice fed a control or low-methionine choline-deficient (LMCD) diet, and injected either with water (W) or diethylnitrosamine (DEN) (n = 8–11 mice per group). **a** PC, **b** polyunsaturated (PU) / saturated (Sat) PC ratio, **c** monounsaturated (MU) / Sat PC ratio, **d** PE, **e** MU-PE, **f** PC / PE ratio. * *P* < 0.05, ** *P* < 0.01, *** *P* < 0.001

The two main lipidomic studies in NASH patients identified a decline of phosphatidylcholine (PC) in the liver [36, 37]. Hepatic PC was also low in the liver of the LMCD diet fed mice, and this decline was significant in the water-injected group (Fig. 4a). In the LMCD model, saturated PC tended to decline and polyunsaturated (PU)-PC was significantly reduced in the liver of water-injected mice (Supplementary Table 5). The PU/saturated PC ratio was not changed by the LMCD diet illustrating that both lipid classes were concordantly regulated (Fig. 4b).

Of note, monounsaturated (MU)-PC increased in the liver of the LMCD diet fed animals, and this effect was significant in the DEN-injected mice (Supplementary Table 5). Accordingly, the MU/saturated PC ratio was found increased in the NASH liver of both groups. Interestingly, this ratio was highest in liver of DEN-injected mice fed the LMCD diet (Fig. 4c).

Total phosphatidylethanolamine (P E) levels were comparable in normal and NASH liver and were not modified by DEN (Fig. 4d). This also applied to PU-PE species (Supplementary Table 5). Saturated PE levels were rather low (< 0.001 nmol/mg), and lipids with a concentration of less than 0.01 nmol/mg were not included in the current calculations. Ratio of -MU-PE to saturated PE was thus not calculated. MU-PE levels were induced in the liver of DEN-injected mice fed the LMCD diet (Fig. 4e), and a similar trend was observed in the liver of the water-injected mice (Fig. 4e). The PC/PE ratio decreases with the progression of NAFLD [14], and also declined in the liver of both animal groups fed the LMCD diet (Fig. 4f). This indicates disturbed membrane integrity in both mouse groups fed the LMCD diet.

**Fig. 5:**
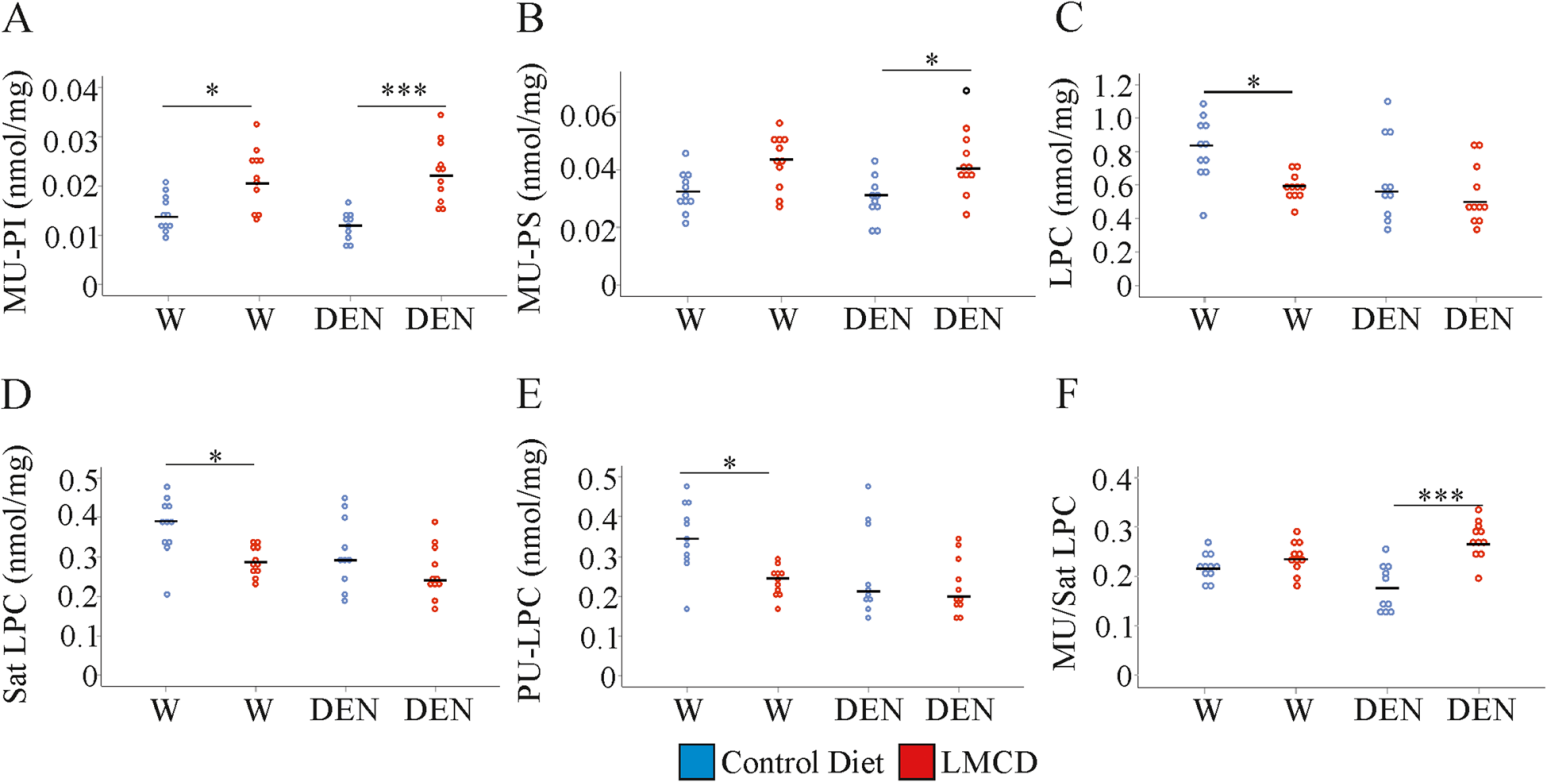
Hepatic phosphatidylinositol (PI), phosphatidylserine (PS) and lysophosphatidylcholine (LPC) levels in mice fed a control or low-methionine choline-deficient (LMCD) diet, and injected either with water (W) or diethylnitrosamine (DEN) (n = 10–11 mice per group). **a** monounsaturated (MU)-PI, **b** MU-PS. **c** LPC, **d** saturated (Sat) LPC, **e** PU-LPC, **f** MU / Sat LPC. * *P* < 0.05, *** *P* < 0.001

Total phosphatidylinositol (PI) levels were not modified by LMCD diet or DEN injection (Supplementary Table 5). MU-PI were higher in NASH liver, and this was significant for both groups (Fig. 5a and Supplementary Table 5). Regarding phosphatidylserine (PS) levels there was a modest increase of MU-PS in the liver of LMCD diet fed animals, which was significant in the DEN-injected mice (Fig. 5b). SCD1 converts saturated to MU fatty acids, but was strongly reduced in the liver of mice fed the LMCD diet [9]. Moreover, in the TG fraction MU species were not increased (Fig. 2c) showing that accumulation of MU phospholipids is achieved by a so far unknown pathway.

### Hepatic lysophosphatidylcholine declines in the liver of water-injected, but not DEN-injected, mice fed the LMCD diet

Lysophosphatidylcholine (LPC) can be generated from PC by phospholipase A2 [38]. The PC level was reduced in NAFLD liver (Fig. 4a) and the LPC level declined accordingly (Fig. 5c). Saturated and PU-LPC were reduced in the NAFLD liver of water-injected mice (Fig. 5d, e and Supplementary Table 5) and MU-LPC showed a similar trend (Supplementary Table 5). PU/saturated LPC and MU/saturated LPC ratios were not altered illustrating concordant changes of saturated, MU and PU LPC species (Fig. 5f and data not shown).

Administration of DEN hindered the decline of total, saturated, and PU-LPC in the liver of the LMCD diet fed mice (Fig. 5c-e). A modest increase of MU-LPC in connection with a small decline of saturated LPC species resulted in a higher MU/saturated LPC ratio (Fig. 5f).

### DEN-injection lowers individual diacylglycerol species in the liver of mice fed the control diet

**Fig. 6:**
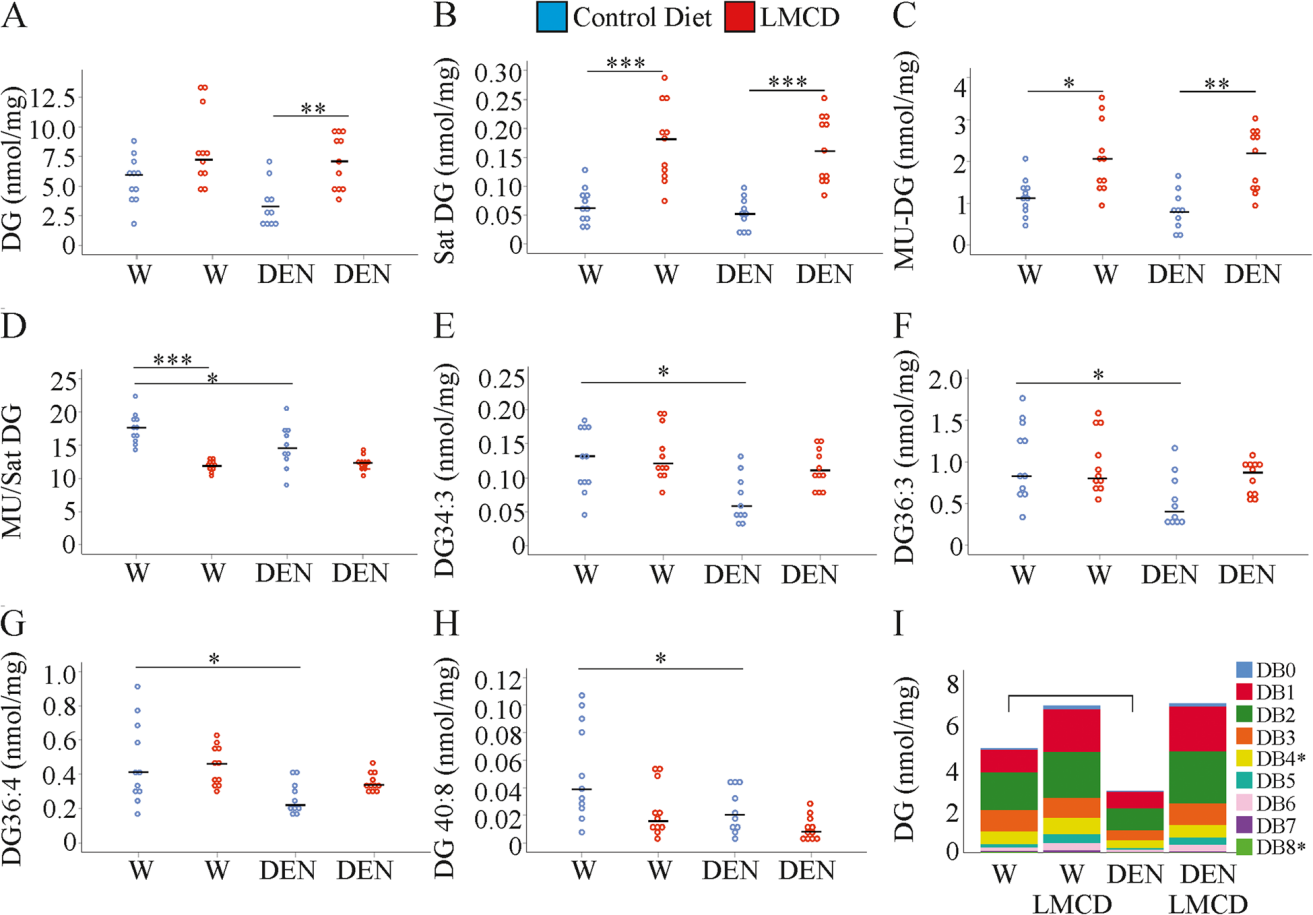
Hepatic diacylglycerol (DG) levels in mice fed a control or low-methionine choline-deficient (LMCD) diet, and injected either with water (W) or diethylnitrosamine (DEN) (n = 10–11 mice per group). **a** DG, **b** saturated (Sat) DG, **c** monounsaturated (MU)-DG, **d** MU / Sat DG ratio, **e** DG 34:3, **f** DG 36:3, **g** DG 36:4, **h** DG 40:8, **i** DG species with increasing number of double bonds. DGs with 4 and 8 double bonds (DB) were lower in the liver of the DEN injected mice fed the control chow. * *P* < 0.05, ** *P* < 0.01, *** *P* < 0.001

Total diacylglycerol (DG) levels had a tendency to be elevated in the liver of water-injected mice and were significantly higher in the DEN group upon feeding a LMCD diet (Fig. 6a). This also applied for PU-DG (Supplementary Table 5). Saturated and MU-DG species were significantly increased in the liver of the LMCD diet fed mice of both groups (Fig. 6b, c). Because of a more prominent upregulation of saturated DGs, the MU/saturated DG ratio declined in liver of the LMCD diet fed mice administered water. A similar regulation, which was not significant, occurred in the DEN treated mice (Fig. 6d).

The MU/saturated DG ratio was already lower in the liver of the DEN-injected mice fed the control diet when compared to the respective water-treated mice (Fig. 6d). This was also evident for the individual species DG 34:3, DG 36:3, 36:4 and DG 40:8 (Fig. 6e-h). When stratified for the number of double bonds DGs with 4 and 8 double bonds declined in the liver of DEN-injected mice fed the control diet (Fig. 6i).

### Ceramides and triacylglycerols decline in HCC tissues

**Fig. 7:**
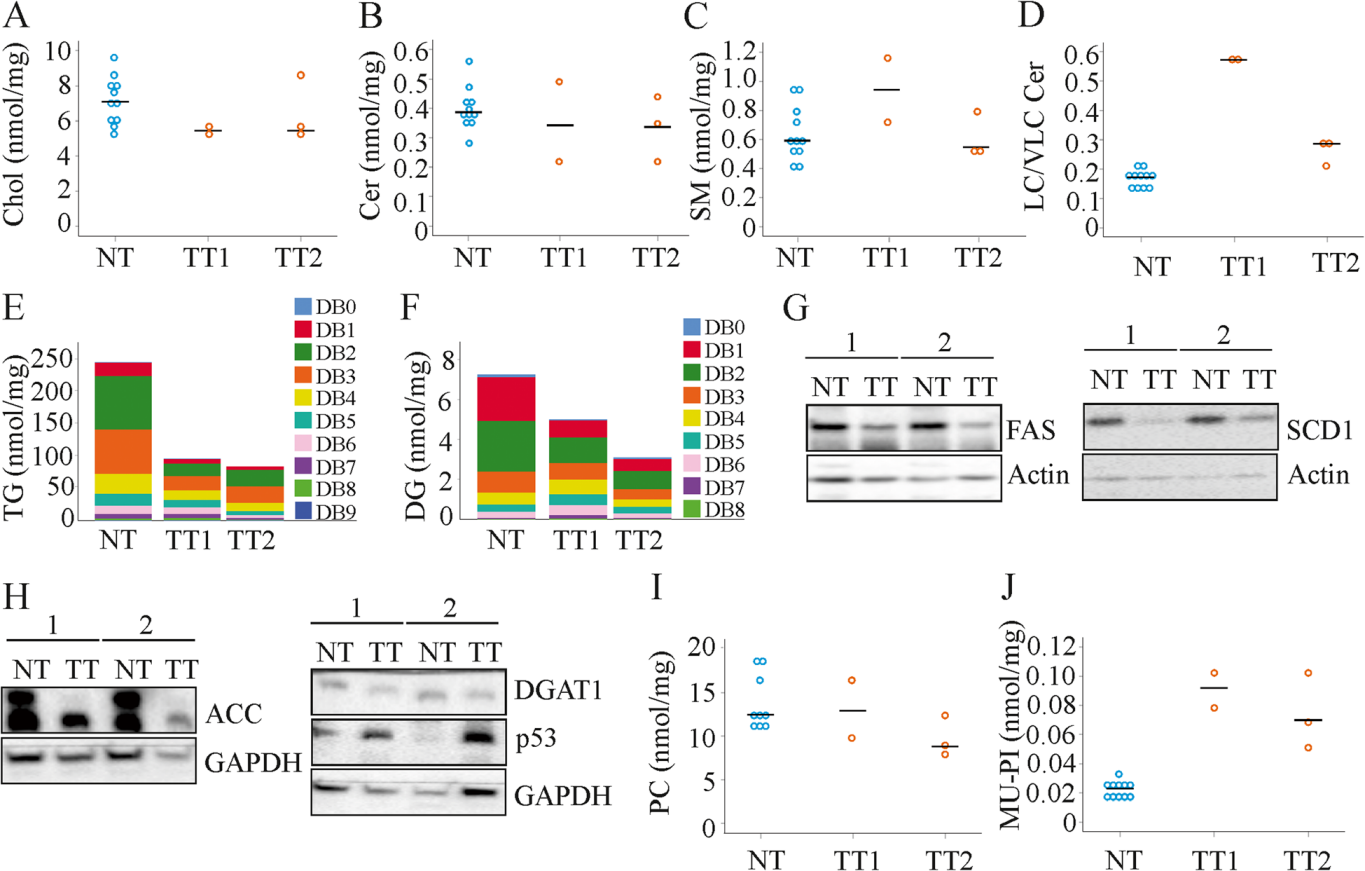
Non-tumor (NT) and tumor (TT) lipids, fatty acid synthase (FAS) and stearoyl-CoA desaturase-1 (SCD1) protein of LMCD diet fed mice (*n* = 5). **a** Cholesterol (Chol), **b** ceramide (Cer), **c** sphingomyelin (SM), **d** long-chain (LC) / very long-chain (VLC) Cer, **e** distribution of triacylglycerols (TG) with increasing number of double bonds (DB) in NT of 9 animals, in two HCCs of one mouse (TT1) and three HCCs of a second mouse (TT2), **f** distribution of diacylglycerols (DG) with increasing number of double bonds in NT of 9 animals, in two HCCs of one mouse (TT1) and three HCCs of a second mouse (TT2), **g** Immunoblot of FAS and SCD1 in NT and TT of two animals, **h** Immunoblot of ACC, DGAT1 and p53 in NT and TT of two animals, **i** phosphatidylcholine (PC), **j** monounsaturated (MU)-phosphatidylinositol (PI)

Hepatocarcinogenesis is linked to lipid reprogramming [11]. Animals injected with DEN developed HCC [9] and five tumors obtained from the liver of two LMCD diet fed mice were large enough for lipid analysis. Total cholesterol and FC were not changed in the murine tumors whereas cholesteryl esters may be reduced (Fig. 7a and Supplementary Table 6).

Decline of ceramides and increase of SM was observed in human HCC tissues [11]. These lipid classes did not differ between non-tumor and tumor tissues in the DEN injected mice fed the LMCD diet (Fig. 7b, c). Of note, very-long chain ceramides, which exert anti-apoptotic functions, even declined in the tumor tissues and the ratio of long to very long chain species was accordingly induced (Fig. 7d). Surprisingly, the sum of TG was low in the cancers, and TGs with any degree of unsaturation were reduced in the tumors (Fig. 7e). Moreover, saturated and unsaturated DGs also declined in the tumors (7f). Consistent with lower TG and DG levels, protein expression of FAS, SCD1 and ACC was suppressed in the tumors (Fig. 7g, h and Table 1). DGAT1 showed a trend to be lower in the tumor tissues (Fig. 7h and Table 1). Considering that fatty acids are essential for cell proliferation, the low expression of proteins involved in fatty acid synthesis in the HCCs was an unexpected finding. The tumor suppressor protein p53 is upregulated in human HCC [39] and was strongly induced in the murine HCC tissues (Fig. 7h and Table 1), and this confirms that the correct tissues have been examined.

**Table 1:** Protein levels of FAS, SCD1, ACC, DGAT1 and p53 in non-tumor (NT) and tumor (TT) tissues of mice fed a LMCD diet and injected with DEN. Number of animals per group (N), median, minimum (Min) and maximum (Max) values and the respective *P* values are listed

Tissue		FAS	SCD1	ACC	DGAT1	p53
NT	N	9	10	6	6	6
	Median	1.10	2.65	1.31	0.91	1.02
	Min	0.49	1.29	0.60	0.51	0.45
	Max	1.96	7.23	2.24	3.09	1.85
TT	N	9	10	5	6	6
	Median	0.46	0.68	0.93	0.79	2.14
	Min	0.17	0.08	0.39	0.39	1.75
	Max	0.92	1.92	0.99	1.32	2.82
	*P*	< 0.001	< 0.001	0.045	0.084	< 0.001

PS, PC and LPC levels did not differ between non-tumorous and HCC tissues (Fig. 7i and Supplementary Table 6). MU-PE and MU-PI, but not PU-PE and PU-PI, were higher in the tumors (Fig. 7j and Supplementary Table 6).

## Discussion

The hepatic lipid profile of the LMCD diet fed mouse resembles human NASH, and was only slightly modified by DEN injection. Still, hepatic oxidative stress and fibrosis grade were not enhanced by DEN. HCC associated lipid reprogramming was not comparable to that of patients, and most strikingly, lipogenesis was even suppressed in the mice. Though this model is unsuitable to test novel drugs that target fatty acid metabolism in HCC, it can be used to clarify the effect of lipogenesis suppression on tumor growth.

Accumulation of TG and DG in the liver is a hallmark of NASH [37, 40], and was also observed in the LMCD diet fed mice. The transcription factor SREBP-1c regulates synthesis of fatty acids [31]. SREBP1c, and its target proteins FAS and SCD1 [31], were strongly suppressed in the LMCD diet induced NASH liver [9]. ACC protein was also reduced in the liver of these mice. From these findings it is concluded that de novo lipogenesis was repressed in the LMCD-NASH liver. DGAT1 catalyzes the final step in TG synthesis [32], and normal DGAT1 protein levels in the LMCD diet fed mouse liver suggest that esterification of fatty acids was not compromised per se. Increased uptake of fatty acids and decreased secretion of lipoprotein particles promoted liver steatosis in the MCD model, and this may also occur in the LMCD diet fed mice [41].

Interestingly, LDL-receptor protein was not reduced in the liver of the LMCD diet fed mice. Expression of the LDL-receptor is among others regulated by SREBP2, which was nevertheless suppressed in the liver of LMCD diet fed mice [9, 31]. Normal hepatic cholesterol levels in the LMCD model suggest that reduced endogenous cholesterol synthesis rather than impaired cholesterol uptake via the LDL-receptor pathway compensates for defective release of lipoproteins into the circulation [41]. FC is a lipotoxic molecule [13] and normal levels in the liver of LMCD fed animals exclude a role of this metabolite in LMCD diet associated hepatic injury.

Oxidative stress is a critical hit in NASH pathogenesis and contributes to hepatic inflammation and fibrosis [10]. Yet, MDA levels and MnSOD expression were normal in the LMCD-NASH liver whereas 4-HNE was strongly increased. MDA and 4-HNE are both produced from PU phospholipids [42]. These metabolites are synthersized by different enzymes and only the pathways enhancing 4-HNE production seemed to be activated in the liver of the LMCD diet fed animals [42].

Traditionally, ceramide was considered as a cytotoxic lipid. Total ceramide content was higher in human NASH liver, and a separate study reported an increase of Cer d18:1/16:0 whereas Cer d18:1/24:0 declined [35, 36]. Of the seven different ceramide species measured all except Cer d18:1/24:1 were increased in the liver of the LMCD diet fed mice. SM levels were quite normal suggesting that ceramide de novo synthesis rather than sphingomyelinases contributed to higher ceramide levels [34]. Blockage of ceramide synthesis improves NASH pathology indicating a functional role of ceramides in disease pathogenesis [43].

A decline of hepatic PC levels was reported in human NASH [36–38]. Hepatic PC was reduced in some murine NASH models [5, 44–47] and was low in the LMCD model studied herein. PC depletion is, however, not mandatory for NASH development and disease progression at least in the murine liver [48]. Moreover, diets deficient in choline do not necessarily cause low hepatic PC [49, 50]. The PC/PE ratio rather than changes in the absolute amounts of PC are highly relevant for membrane integrity and liver health [51]. The hepatic PC/PE ratio was reduced in human NASH [52] and in the LMCD model.

A decline of systemic LPC in mice and patients with chronic liver diseases was already reported [53–56]. Moreover, the decline of hepatic PC may cause lower LPC levels in NASH liver. Though low LPC levels were indeed observed in the current model, Puri et al. described higher LPC in human NASH liver [37]. Murine studies identified increased and unchanged LPC in NASH liver [5, 37, 38]. Thus, the source and regulation of hepatic LPC in NASH remains unknown. Evaluation of these pathways may also clarify why LMCD diet associated decline of hepatic LPC was abolished in the DEN treated mice.

The ratio of MU/saturated PC was higher in the NASH liver of DEN-injected than control-treated mice. Oleate is the most abundant intracellular monounsaturated fatty acid [57]. Oleate indeed downregulated tumor suppressor molecules and enhanced colony formation in an in-vitro model [58]. Such an effect was not observed with palmitate [58], and dietary saturated fat even delayed initiation of HCC in the DEN model [59]. A diet high in saturated fat retarded carcinogenesis by DEN in rats in comparison to rats fed a diet enriched in PU fatty acids [60]. Thus, DEN associated increase of monounsaturated fatty acids in the PC fraction may promote hepatocarcinogenesis. The pathways which contribute to higher MU phospholipids have yet to be identified. SCD1 expression [9] was comparable in the liver of water and DEN injected mice fed the LMCD diet. Cells may use alternative pathways to increase levels of MU fatty acids and enzymes like fatty acid desaturase 2 [61] may contribute to higher MU lipid concentrations in the DEN treated mice.

DEN injection was further associated with reduced levels of several DG species in the liver of control diet fed mice. DGs are signalling molecules that activate protein kinase C. Nowadays, diacylglycerol-sensitive protein kinase C isoenzymes are regarded as tumor suppressors [62]. Therefore, it deserves further investigations to evaluate the role of individual DG species in hepatic carcinogenesis.

Mice fed the LMCD diet and injected with DEN developed HCC. The tumor suppressor protein p53 was strongly induced in the tumors and this was shown before in patients with non-viral HCC [39]. Most of the tumors were small and only five tumors obtained from the liver of two mice were suitable for lipid analysis. DEN injected mice fed the control chow had fewer tumors [9], and only 2 tumors of one animal were appropriate for lipidomic analysis. Thus, it was not possible to compare the lipid composition of LMCD diet and control chow fed mice.

Just a few lipids were changed in the tumors of the LMCD-HCC model. The consistently reported decline of ceramide and PU-phospholipids in HCC was not observed [11]. Li et al. showed that phospholipids with 0, 1 or 3 double bonds were upregulated whereas lipids with 2, 4 or 6 double bonds were down-regulated in HCC tissues [63]. MU-PE and MU-PI were indeed increased in the tumors of the DEN-LMCD mice but a concurrent decline of PU-lipids was not observed. Enhanced lipogenesis in human HCC promotes the increase of saturated fats at the expense of of PU lipids [11]. Lipogenesis was even suppressed in murine HCC and PU-lipids did not decline. The pathophysiological roles of the phospholipid derangements described above in the progression of cancerogenesis are not well studied. Whether the decline in PU phospholipids observed in human HCCs is indeed a main driver of tumor progression and represents a pathophysiological relevant difference between human and murine HCCs requires further investigation.

In the majority of human HCCs lipogenesis and TG levels were greatly enhanced [64, 65]. In the LMCD diet fed animals TG and DG levels even declined in the HCC tissues. Accordingly, FAS, SCD1 and ACC were suppressed in the tumors and DGAT1 tended to decline.

Not all murine and human HCCs overexpress FAS [64, 66] and uptake of exogenous fatty acids can also support HCC growth [65]. Inhibitors of lipogenesis may be effective drugs only in liver tumors with high endogenous fatty acid synthesis. One study even showed that blockage of ACC increased the susceptibility to DEN initiated tumorigenesis. Tumor cells acquired an antioxidant defence and had increased survival [67]. These findings reveal a knowledge gap concerning the pathogenic role of lipogenesis in HCC [67]. The LMCD-HCC model may be useful to study liver tumorigenesis when endogenous lipid synthesis is blocked.

Effects of drugs that target ACC or FAS in HCC can, however, not be analysed in this model. Recent [67] and present findings suggest that inhibitors of lipogenesis should be used with caution in HCC therapy.

Indeed, HCC is a very heterogeneous disease which was mostly described at the molecular level in human tissues so far [68]. There are emerging data showing significant inter-tumor differences in fatty acid metabolism of human HCCs [64]. To study the various aspects of the human disease different murine HCC models have to be employed.

Restoration of ceramide by nanoliposomal C6-ceramide containing particles represents an additional option to treat HCC. Though various studies indicated a role of lipogenesis and ceramide in HCC pathogenesis, their pathophysiological and therapeutic value was not finally clarified [11].

### Study strengths and limitations

The strength of this study was the analysis of various lipid species in the liver of the LMCD diet fed mice, which is a better suited NASH model than the MCD diet fed mice. Moreover, the DEN injection associated changes in distinct lipid classes were identified. Limitation of the study was that lipids of only five tumors resected from two animals were analysed. Notably, TG and DG levels were reduced in the tumors. Lipogenic enzymes could be quantified in the liver of up to ten mice per group, and low expression in the tumors also suggested that lipogenesis was suppressed. Therefore, additional studies need to be undertaken in order to better characterize the cancer lipidome of the LMCD model in the context of NASH and HCC.

## Conclusions

The LMCD fed mice are suited to study the pathophysiological role of lipid derangements observed in a subgroup of NASH patients. Lipids may become targets for NASH therapies, and the respective drugs can the tested in this preclinical model. Since there are no approved drugs for the treatment of NASH this approach is relevant prior to their use in a clinical setting. Preliminary data indicated that the HCC related lipidome did not resemble the lipid derangements reported in tissues of HCC patients. Drugs that suppress lipogenesis in HCC are believed to reduce tumor growth. However, the observation that lipogenesis is downregulated in the tumors of the LMCD diet fed mice, which still develop HCC may question the clinical relevance of such an assumption. Therefore, the LMCD-HCC model can be used to clarify the adverse effects of such therapies.

## Supplementary Information

**Additional file 1: Table S1.** Hepatic proteins in mice fed a control or low-methionine choline-deficient (LMCD) diet, and injected either with water (W) or diethylnitrosamine (DEN) (*n* = 5–11 mice per group). GAPDH was used for normalization except for MnSOD, which was normalized to the Coomassie Blue stained protein levels. Median, minimum (Min) and maximum (Max) values are listed. The *P* values for comparison of water injected mice (*P*_water_) and DEN injected mice (*P*_DEN_) fed the control chow or LMCD diet are listed. Not significant, ns. **Table S2.** Levels of triacylglycerol (TG) species are given in nmol/mg. Median value, minimum (Min) and maximum (Max) are listed. The *P* values for comparison of water (*P*_Water_) and DEN injected mice (*P*_DEN_) fed the control chow or LMCD diet are listed. Comparison of the mouse groups fed the LMCD diet identified two differentially abundant TG species (*P*_LMCD_). Comparison of the mouse groups fed the control chow did not identify any differences (data not shown). Not significant, ns. **Table S3.** Proportion of triacylglycerol (TG) species in % of total TG. Median value, minimum (Min) and maximum (Max) of % TG are given. The *P* values for comparison of water injected mice and DEN injected mice fed the control chow are listed. Comparison of water injected mice and DEN injected mice fed the LMCD diet did not identify significant differences. The arrow indicates whether the respective TG is higher or lower in the liver of the DEN injected mouse. **Table S4.** Levels of free cholesterol (FC), cholesteryl ester (CE) and total cholesterol are given in nmol/mg. Median value, minimum (Min) and maximum (Max) are listed. The *P* values for comparison of water injected mice (*P*_Water_) and DEN injected mice (*P*_DEN_) fed the control chow or LMCD diet are listed. Comparison of the mice groups fed the control diet (P_Control diet_) or LMCD diet (P_LMCD_) is also given. Not significant, ns. **Table S5.** Levels of diacylglycerol (DG), phosphatidylcholine (PC), phosphatidylethanolamine (PE), lysophosphatidylcholine (LPC), phosphatidylserine (PS) and phosphatidylinositol (PI) are given in nmol/mg. Median value, minimum (Min) and maximum (Max) are listed. The *P* values for comparison of water injected mice (*P*_Water_) and DEN injected mice (*P*_DEN_) fed the control chow or LMCD diet are listed. *P* values for the comparison of the mice groups fed the control diet (P_Control diet_) is also given. Comparison of water injected mice and DEN injected mice fed the LMCD diet did not identify significant differences. Not significant, ns; Sat, saturated; MU, monounsaturated; PU, polyunsaturated. **Table S6.** Lipidome of normal liver and tumor tissues of two mice (1, 2). Levels of free cholesterol (FC), cholesteryl ester (CE), phosphatidylcholine (PC), phosphatidylethanolamine (PE), lysophosphatidylcholine (LPC), phosphatidylserine (PS) and phosphatidylinositol (PI) are given in nmol/mg. Median value, minimum (Min) and maximum (Max) are listed. % change in the tumors is given in the final two columns. Phospholipid, PL.

## Data Availability

The datasets generated and/or analyzed during the current study are available from the corresponding author on reasonable request.

## Abbreviations

4-HNE: 4-Hydroxynonenal
ACC: Acetyl-CoA-carboxylase
AFP: Alpha-fetoprotein
Cer: Ceramide
CE: Cholesteryl ester
DG: Diacylglycerol
DGAT1: Diacylglycerol-O-acyltransferase 1
FAS: Fatty Acid Synthase
LDL: Low density lipoprotein
LPC: Lysophosphatidylcholine
MDA: Malondialdehyde
MnSOD: Manganese Superoxide Dismutase
PC: Phosphatidylcholine
SM: Sphingomyelin
SCD1: Stearoyl-CoA Desaturase-1
TG: Triacylglycerol

## References

[1] Berlanga A, Guiu-Jurado E, Porras JA, Auguet T (2014). Molecular pathways in non-alcoholic fatty liver disease. Clin Exp Gastroenterol.

[2] Buechler C, Wanninger J, Neumeier M (2011). Adiponectin, a key adipokine in obesity related liver diseases. World J Gastroenterol.

[3] Schaffler A, Scholmerich J, Buchler C (2005). Mechanisms of disease: adipocytokines and visceral adipose tissue--emerging role in nonalcoholic fatty liver disease. Nat Clin Pract Gastroenterol Hepatol.

[4] Schattenberg JM, Galle PR (2010). Animal models of non-alcoholic steatohepatitis: of mice and man. Dig Dis.

[5] Rein-Fischboeck L, Haberl EM, Pohl R, Feder S, Liebisch G, Krautbauer S, Buechler C (2019). Variations in hepatic lipid species of age-matched male mice fed a methionine-choline-deficient diet and housed in different animal facilities. Lipids Health Dis.

[6] Rein-Fischboeck L, Haberl EM, Pohl R, Schmid V, Feder S, Krautbauer S, Liebisch G, Buechler C (1863). Alpha-syntrophin null mice are protected from non-alcoholic steatohepatitis in the methionine-choline-deficient diet model but not the atherogenic diet model. Biochim Biophys Acta.

[7] Bakiri L, Wagner EF (2013). Mouse models for liver cancer. Mol Oncol.

[8] Matsumoto M, Hada N, Sakamaki Y, Uno A, Shiga T, Tanaka C, Ito T, Katsume A, Sudoh M (2013). An improved mouse model that rapidly develops fibrosis in non-alcoholic steatohepatitis. Int J Exp Pathol.

[9] Haberl EM, Pohl R, Rein-Fischboeck L, Feder S, Sinal CJ, Buechler C (2018). Chemerin in a mouse model of non-alcoholic Steatohepatitis and Hepatocarcinogenesis. Anticancer Res.

[10] Neuschwander-Tetri BA (2010). Hepatic lipotoxicity and the pathogenesis of nonalcoholic steatohepatitis: the central role of nontriglyceride fatty acid metabolites. Hepatology..

[11] Buechler C, Aslanidis C (2020). Role of lipids in pathophysiology, diagnosis and therapy of hepatocellular carcinoma. Biochim Biophys Acta Mol Cell Biol Lipids.

[12] Montgomery MK, Brown SH, Lim XY, Fiveash CE, Osborne B, Bentley NL, Braude JP, Mitchell TW, Coster AC, Don AS (2016). Regulation of glucose homeostasis and insulin action by ceramide acyl-chain length: a beneficial role for very long-chain sphingolipid species. Biochim Biophys Acta.

[13] Ioannou GN (2016). The role of cholesterol in the pathogenesis of NASH. Trends Endocrinol Metab.

[14] Cano A, Alonso C (2014). Deciphering non-alcoholic fatty liver disease through metabolomics. Biochem Soc Trans.

[15] Giudetti AM, De Domenico S, Ragusa A, Lunetti P, Gaballo A, Franck J, Simeone P, Nicolardi G, De Nuccio F, Santino A (2019). A specific lipid metabolic profile is associated with the epithelial mesenchymal transition program. Biochim Biophys Acta Mol Cell Biol Lipids.

[16] Garattini S, Grignaschi G (2017). Animal testing is still the best way to find new treatments for patients. Eur J Intern Med.

[17] Liu M, Yan Q, Sun Y, Nam Y, Hu L, Loong JH, Ouyang Q, Zhang Y, Li HL, Kong FE (2020). A hepatocyte differentiation model reveals two subtypes of liver cancer with different oncofetal properties and therapeutic targets. Proc Natl Acad Sci U S A.

[18] Liebisch G, Binder M, Schifferer R, Langmann T, Schulz B, Schmitz G (1761). High throughput quantification of cholesterol and cholesteryl ester by electrospray ionization tandem mass spectrometry (ESI-MS/MS). Biochim Biophys Acta.

[19] Liebisch G, Lieser B, Rathenberg J, Drobnik W, Schmitz G (2004). High-throughput quantification of phosphatidylcholine and sphingomyelin by electrospray ionization tandem mass spectrometry coupled with isotope correction algorithm. Biochim Biophys Acta.

[20] Liebisch G, Drobnik W, Lieser B, Schmitz G (2002). High-throughput quantification of lysophosphatidylcholine by electrospray ionization tandem mass spectrometry. Clin Chem.

[21] Matyash V, Liebisch G, Kurzchalia TV, Shevchenko A, Schwudke D (2008). Lipid extraction by methyl-tert-butyl ether for high-throughput lipidomics. J Lipid Res.

[22] Zemski Berry KA, Murphy RC (2004). Electrospray ionization tandem mass spectrometry of glycerophosphoethanolamine plasmalogen phospholipids. J Am Soc Mass Spectrom.

[23] Liebisch G, Drobnik W, Reil M, Trumbach B, Arnecke R, Olgemoller B, Roscher A, Schmitz G (1999). Quantitative measurement of different ceramide species from crude cellular extracts by electrospray ionization tandem mass spectrometry (ESI-MS/MS). J Lipid Res.

[24] Horing M, Ejsing CS, Hermansson M, Liebisch G (2019). Quantification of cholesterol and Cholesteryl Ester by direct flow injection high-resolution Fourier transform mass spectrometry utilizing species-specific response factors. Anal Chem.

[25] Husen P, Tarasov K, Katafiasz M, Sokol E, Vogt J, Baumgart J, Nitsch R, Ekroos K, Ejsing CS (2013). Analysis of lipid experiments (ALEX): a software framework for analysis of high-resolution shotgun lipidomics data. PLoS One.

[26] Horing M, Ekroos K, Baker PRS, Connell L, Stadler SC, Burkhardt R, Liebisch G (2020). Correction of isobaric overlap resulting from sodiated ions in lipidomics. Anal Chem.

[27] Liebisch G, Vizcaino JA, Kofeler H, Trotzmuller M, Griffiths WJ, Schmitz G, Spener F, Wakelam MJ (2013). Shorthand notation for lipid structures derived from mass spectrometry. J Lipid Res.

[28] Bligh EG, Dyer WJ (1959). A rapid method of total lipid extraction and purification. Can J Biochem Physiol.

[29] Schneider CA, Rasband WS, Eliceiri KW (2012). NIH image to ImageJ: 25 years of image analysis. Nat Methods.

[30] Beneduce L, Castaldi F, Marino M, Tono N, Gatta A, Pontisso P, Fassina G (2004). Improvement of liver cancer detection with simultaneous assessment of circulating levels of free alpha-fetoprotein (AFP) and AFP-IgM complexes. Int J Biol Markers.

[31] Horton JD (2002). Sterol regulatory element-binding proteins: transcriptional activators of lipid synthesis. Biochem Soc Trans.

[32] Millar JS, Stone SJ, Tietge UJ, Tow B, Billheimer JT, Wong JS, Hamilton RL, Farese RV, Rader DJ (2006). Short-term overexpression of DGAT1 or DGAT2 increases hepatic triglyceride but not VLDL triglyceride or apoB production. J Lipid Res.

[33] Luukkonen PK, Zhou Y, Sadevirta S, Leivonen M, Arola J, Oresic M, Hyotylainen T, Yki-Jarvinen H (2016). Hepatic ceramides dissociate steatosis and insulin resistance in patients with non-alcoholic fatty liver disease. J Hepatol.

[34] Gault CR, Obeid LM, Hannun YA (2010). An overview of sphingolipid metabolism: from synthesis to breakdown. Adv Exp Med Biol.

[35] Apostolopoulou M, Gordillo R, Koliaki C, Gancheva S, Jelenik T, De Filippo E, Herder C, Markgraf D, Jankowiak F, Esposito I (2018). Specific hepatic Sphingolipids relate to insulin resistance, oxidative stress, and inflammation in nonalcoholic Steatohepatitis. Diabetes Care.

[36] Chiappini F, Coilly A, Kadar H, Gual P, Tran A, Desterke C, Samuel D, Duclos-Vallee JC, Touboul D, Bertrand-Michel J (2017). Metabolism dysregulation induces a specific lipid signature of nonalcoholic steatohepatitis in patients. Sci Rep.

[37] Puri P, Baillie RA, Wiest MM, Mirshahi F, Choudhury J, Cheung O, Sargeant C, Contos MJ, Sanyal AJ (2007). A lipidomic analysis of nonalcoholic fatty liver disease. Hepatology..

[38] Musso G, Cassader M, Paschetta E, Gambino R (2018). Bioactive lipid species and metabolic pathways in progression and resolution of nonalcoholic Steatohepatitis. Gastroenterology.

[39] Krautbauer S, Meier EM, Rein-Fischboeck L, Pohl R, Weiss TS, Sigruener A, Aslanidis C, Liebisch G, Buechler C (2016). Ceramide and polyunsaturated phospholipids are strongly reduced in human hepatocellular carcinoma. Biochim Biophys Acta.

[40] Gorden DL, Ivanova PT, Myers DS, McIntyre JO, VanSaun MN, Wright JK, Matrisian LM, Brown HA (2011). Increased diacylglycerols characterize hepatic lipid changes in progression of human nonalcoholic fatty liver disease; comparison to a murine model. PLoS One.

[41] Caballero F, Fernandez A, Matias N, Martinez L, Fucho R, Elena M, Caballeria J, Morales A, Fernandez-Checa JC, Garcia-Ruiz C (2010). Specific contribution of methionine and choline in nutritional nonalcoholic steatohepatitis: impact on mitochondrial S-adenosyl-L-methionine and glutathione. J Biol Chem.

[42] Ayala A, Munoz MF, Arguelles S (2014). Lipid peroxidation: production, metabolism, and signaling mechanisms of malondialdehyde and 4-hydroxy-2-nonenal. Oxidative Med Cell Longev.

[43] Kasumov T, Li L, Li M, Gulshan K, Kirwan JP, Liu X, Previs S, Willard B, Smith JD, McCullough A (2015). Ceramide as a mediator of non-alcoholic fatty liver disease and associated atherosclerosis. PLoS One.

[44] Eisinger K, Krautbauer S, Hebel T, Schmitz G, Aslanidis C, Liebisch G, Buechler C (2014). Lipidomic analysis of the liver from high-fat diet induced obese mice identifies changes in multiple lipid classes. Exp Mol Pathol.

[45] Laggai S, Simon Y, Ranssweiler T, Kiemer AK, Kessler SM (2013). Rapid chromatographic method to decipher distinct alterations in lipid classes in NAFLD/NASH. World J Hepatol.

[46] Mas E, Danjoux M, Garcia V, Carpentier S, Segui B, Levade T (2009). IL-6 deficiency attenuates murine diet-induced non-alcoholic steatohepatitis. PLoS One.

[47] Najt CP, Senthivinayagam S, Aljazi MB, Fader KA, Olenic SD, Brock JR, Lydic TA, Jones AD, Atshaves BP (2016). Liver-specific loss of Perilipin 2 alleviates diet-induced hepatic steatosis, inflammation, and fibrosis. Am J Physiol Gastrointest Liver Physiol.

[48] Niebergall LJ, Jacobs RL, Chaba T, Vance DE (2011). Phosphatidylcholine protects against steatosis in mice but not non-alcoholic steatohepatitis. Biochim Biophys Acta.

[49] Cui Z, Houweling M, Vance DE (1995). Expression of phosphatidylethanolamine N-methyltransferase-2 in McArdle-RH7777 hepatoma cells inhibits the CDP-choline pathway for phosphatidylcholine biosynthesis via decreased gene expression of CTP:phosphocholine cytidylyltransferase. Biochem J.

[50] Kulinski A, Vance DE, Vance JE (2004). A choline-deficient diet in mice inhibits neither the CDP-choline pathway for phosphatidylcholine synthesis in hepatocytes nor apolipoprotein B secretion. J Biol Chem.

[51] Li Z, Agellon LB, Allen TM, Umeda M, Jewell L, Mason A, Vance DE (2006). The ratio of phosphatidylcholine to phosphatidylethanolamine influences membrane integrity and steatohepatitis. Cell Metab.

[52] Arendt BM, Ma DW, Simons B, Noureldin SA, Therapondos G, Guindi M, Sherman M, Allard JP (2013). Nonalcoholic fatty liver disease is associated with lower hepatic and erythrocyte ratios of phosphatidylcholine to phosphatidylethanolamine. Appl Physiol Nutr Metab.

[53] Eisinger K, Liebisch G, Schmitz G, Aslanidis C, Krautbauer S, Buechler C (2014). Lipidomic analysis of serum from high fat diet induced obese mice. Int J Mol Sci.

[54] Patterson AD, Maurhofer O, Beyoglu D, Lanz C, Krausz KW, Pabst T, Gonzalez FJ, Dufour JF, Idle JR (2011). Aberrant lipid metabolism in hepatocellular carcinoma revealed by plasma metabolomics and lipid profiling. Cancer Res.

[55] Tanaka N, Matsubara T, Krausz KW, Patterson AD, Gonzalez FJ (2012). Disruption of phospholipid and bile acid homeostasis in mice with nonalcoholic steatohepatitis. Hepatology..

[56] Zhou L, Ding L, Yin P, Lu X, Wang X, Niu J, Gao P, Xu G (2012). Serum metabolic profiling study of hepatocellular carcinoma infected with hepatitis B or hepatitis C virus by using liquid chromatography-mass spectrometry. J Proteome Res.

[57] Ackerman D, Tumanov S, Qiu B, Michalopoulou E, Spata M, Azzam A, Xie H, Simon MC, Kamphorst JJ (2018). Triglycerides promote lipid homeostasis during hypoxic stress by balancing fatty acid saturation. Cell Rep.

[58] Kudo Y, Tanaka Y, Tateishi K, Yamamoto K, Yamamoto S, Mohri D, Isomura Y, Seto M, Nakagawa H, Asaoka Y (2011). Altered composition of fatty acids exacerbates hepatotumorigenesis during activation of the phosphatidylinositol 3-kinase pathway. J Hepatol.

[59] Duan XY, Pan Q, Yan SY, Ding WJ, Fan JG, Qiao L (2014). High-saturate-fat diet delays initiation of diethylnitrosamine-induced hepatocellular carcinoma. BMC Gastroenterol.

[60] Glauert HP, Lay LT, Kennan WS, Pitot HC (1991). Effect of dietary fat on the initiation of hepatocarcinogenesis by diethylnitrosamine or 2-acetylaminofluorene in rats. Carcinogenesis.

[61] Vriens K, Christen S, Parik S, Broekaert D, Yoshinaga K, Talebi A, Dehairs J, Escalona-Noguero C, Schmieder R, Cornfield T (2019). Evidence for an alternative fatty acid desaturation pathway increasing cancer plasticity. Nature..

[62] Newton AC (2018). Protein kinase C: perfectly balanced. Crit Rev Biochem Mol Biol.

[63] Li Z, Guan M, Lin Y, Cui X, Zhang Y, Zhao Z, Zhu J. Aberrant lipid metabolism in hepatocellular carcinoma revealed by liver Lipidomics. Int J Mol Sci. 2017;18:2550. 10.3390/ijms18122550 PMC5751153 29182572

[64] Berndt N, Eckstein J, Heucke N, Gajowski R, Stockmann M, Meierhofer D, Holzhutter HG. Characterization of lipid and lipid droplet metabolism in human HCC. Cells. 2019;8:512. 10.3390/cells8050512 PMC6562484 31137921

[65] De Matteis S, Ragusa A, Marisi G, De Domenico S, Casadei Gardini A, Bonafe M, Giudetti AM (2018). Aberrant metabolism in hepatocellular carcinoma provides diagnostic and therapeutic opportunities. Oxidative Med Cell Longev.

[66] Che L, Pilo MG, Cigliano A, Latte G, Simile MM, Ribback S, Dombrowski F, Evert M, Chen X, Calvisi DF (2017). Oncogene dependent requirement of fatty acid synthase in hepatocellular carcinoma. Cell Cycle.

[67] Nelson ME, Lahiri S, Chow JD, Byrne FL, Hargett SR, Breen DS, Olzomer EM, Wu LE, Cooney GJ, Turner N (2017). Inhibition of hepatic lipogenesis enhances liver tumorigenesis by increasing antioxidant defence and promoting cell survival. Nat Commun.

[68] Jovel J, Lin Z, O'Keefe S, Willows S, Wang W, Zhang G, Patterson J, Moctezuma-Velazquez C, Kelvin DJ, Ka-Shu Wong G, Mason AL (2018). A survey of molecular heterogeneity in hepatocellular carcinoma. Hepatol Commun.

