# Supplementary material for: Hepatic lipid profile in mice fed a choline-deficient, low-methionine diet resembles human non-alcoholic fatty liver disease
Source: Lipids Health Dis. 2020 Dec 9;19:250. doi: 10.1186/s12944-020-01425-1 (PMC7727224; doi:10.1186/s12944-020-01425-1)
Supplement: Supplementary file 1 — Additional file 1: Table S1. Hepatic proteins in mice fed a control or low-methionine choline-deficient (LMCD) diet, and injected either with water (W) or diethylnitrosamine (DEN) (n = 5–11 mice per group). GAPDH was used for normalization except for MnSOD, which was normalized to the Coomassie Blue stained protein levels. Median, minimum (Min) and maximum (Max) values are listed. The P values for comparison of water injected mice (Pwater) and DEN injected mice (PDEN) fed the control chow or LMCD diet are listed. Not significant, ns. Table S2. Levels of triacylglycerol (TG) species are given in nmol/mg. Median value, minimum (Min) and maximum (Max) are listed. The P values for comparison of water (PWater) and DEN injected mice (PDEN) fed the control chow or LMCD diet are listed. Comparison of the mouse groups fed the LMCD diet identified two differentially abundant TG species (PLMCD). Comparison of the mouse groups fed the control chow did not identify any differences (data not shown). Not significant, ns. Table S3. Proportion of triacylglycerol (TG) species in % of total TG. Median value, minimum (Min) and maximum (Max) of % TG are given. The P values for comparison of water injected mice and DEN injected mice fed the control chow are listed. Comparison of water injected mice and DEN injected mice fed the LMCD diet did not identify significant differences. The arrow indicates whether the respective TG is higher or lower in the liver of the DEN injected mouse. Table S4. Levels of free cholesterol (FC), cholesteryl ester (CE) and total cholesterol are given in nmol/mg. Median value, minimum (Min) and maximum (Max) are listed. The P values for comparison of water injected mice (PWater) and DEN injected mice (PDEN) fed the control chow or LMCD diet are listed. Comparison of the mice groups fed the control diet (PControl diet) or LMCD diet (PLMCD) is also given. Not significant, ns. Table S5. Levels of diacylglycerol (DG), phosphatidylcholine (PC), phosphatidylethanolam [file 12944_2020_1425_MOESM1_ESM.docx]

Supplementary Table 1: Hepatic proteins in mice fed a control or low-methionine choline-deficient (LMCD) diet, and injected either with water (W) or diethylnitrosamine (DEN) (n = 5 - 11 mice per group). GAPDH was used for normalization except for MnSOD, which was normalized to the Coomassie Blue stained protein levels. Median, minimum (Min) and maximum (Max) values are listed. The *P* values for comparison of water injected mice (*P*_water_) and DEN injected mice (*P*_DEN_) fed the control chow or LMCD diet are listed. Not significant, ns.

|  | **Water** | | | | | **LMCD Water** | | | ***P***_Water_ | **DEN** | | | | **LMCD DEN** | | | ***P***_DEN_ |
| --- | --- | --- | --- | --- | --- | --- | --- | --- | --- | --- | --- | --- | --- | --- | --- | --- | --- |
| Protein | Median | Min | | Max | Median | | Min | Max |  | Median | Min | Max | Median | | Min | Max |  |
| Col1A1 | 0.9 | 0.2 | 1.9 | | 3.0 | | 1.1 | 4.2 | 0.023 | 1.2 | 0.7 | 3.3 | 3.8 | | 1.9 | 0.3 | 0.014 |
| Alpha-SMA | 0.1 | 0 | 0.1 | | 0.3 | | 0.2 | 1.0 | 0.036 | 0.1 | 0 | 0.12 | 0.5 | | 0.3 | 0.8 | 0.019 |
| MnSOD | 0.6 | 0.1 | 1.0 | | 0.6 | | 0.3 | 0.9 | ns | 0.5 | 0.4 | 0.8 | 0.6 | | 0.2 | 0.8 | ns |
| 4-HNE | 1.1 | 0.2 | 1. 9 | | 5.1 | | 3.1 | 7. 7 | 0.001 | 1.6 | 0.1 | 3.9 | 4.4 | | 2.6 | 5.6 | 0.045 |
| ACC | 2.0 | 0.7 | 3.4 | | 0.8 | | 0.3 | 1.4 | 0.034 | 1.6 | 0.6 | 2.2 | 0.6 | | 0.4 | 0.9 | ns |
| DGAT1 | 0.7 | 0.7 | 1.2 | | 1.0 | | 0.6 | 1.5 | ns | 0.9 | 0.4 | 1.0 | 0.6 | | 0.5 | 0.6 | ns |
| FABP5 | 1.7 | 0.8 | 2.6 | | 0.5 | | 0.3 | 0.6 | < 0.001 | 1.4 | 1.3 | 2.2 | 0.5 | | 0.4 | 0.8 | 0.001 |
| LDL-R | 1.9 | 0.4 | 3.1 | | 1.5 | | 0.8 | 2.4 | ns | 1.8 | 0.9 | 4.2 | 1.1 | | 0.7 | 3.0 | ns |

Supplementary Table 2: Levels of triacylglycerol (TG) species are given in nmol/mg. Median value, minimum (Min) and maximum (Max) are listed. The *P* values for comparison of water (*P*_Water_) and DEN injected mice (*P*_DEN_) fed the control chow or LMCD diet are listed. Comparison of the mouse groups fed the LMCD diet identified two differentially abundant TG species (*P*_LMCD_). Comparison of the mouse groups fed the control chow did not identify any differences (data not shown). Not significant, ns.

|  | **Water** | | | **LMCD Water** | | | **DEN** | | | **LMCD DEN** | | | ***P***_Water_ | ***P***_DEN_ | ***P*** _LMCD_ |
| --- | --- | --- | --- | --- | --- | --- | --- | --- | --- | --- | --- | --- | --- | --- | --- |
| TG | Median | Min | Max | Median | Min | Max | Median | Min | Max | Median | Min | Max |  |  |  |
| 46:0 | 0.015 | 0.008 | 0.053 | 0.353 | 0.083 | 0.568 | 0.023 | 0.002 | 0.042 | 0.256 | 0.071 | 0.415 | <0.001 | <0.001 | ns |
| 46:1 | 0.039 | 0.023 | 0.206 | 2.889 | 1.350 | 4.415 | 0.042 | 0.010 | 0.093 | 2.412 | 1.308 | 3.522 | <0.001 | <0.001 | ns |
| 46:2 | 0.026 | 0.012 | 0.239 | 1.435 | 0.989 | 2.498 | 0.020 | 0.015 | 0.046 | 1.297 | 0.810 | 1.917 | <0.001 | <0.001 | ns |
| 48:0 | 0.103 | 0.061 | 0.311 | 0.663 | 0.317 | 1.396 | 0.141 | 0.014 | 0.252 | 0.535 | 0.000 | 0.955 | <0.001 | ns | ns |
| 48:1 | 0.440 | 0.253 | 1.408 | 5.179 | 2.216 | 8.143 | 0.541 | 0.066 | 1.221 | 4.410 | 2.571 | 6.431 | <0.001 | <0.001 | ns |
| 48:2 | 0.328 | 0.199 | 1.032 | 4.836 | 2.222 | 6.967 | 0.350 | 0.095 | 0.842 | 3.900 | 2.459 | 6.037 | <0.001 | <0.001 | ns |
| 48:3 | 0.092 | 0.049 | 0.398 | 1.546 | 0.988 | 2.364 | 0.083 | 0.065 | 0.183 | 1.348 | 0.838 | 1.979 | <0.001 | <0.001 | ns |
| 48:4 | 0.060 | 0.039 | 0.122 | 0.440 | 0.324 | 0.677 | 0.063 | 0.035 | 0.087 | 0.354 | 0.239 | 0.575 | <0.001 | <0.001 | ns |
| 49:1 | 0.027 | 0.015 | 0.077 | 0.184 | 0.079 | 0.332 | 0.030 | 0.002 | 0.072 | 0.143 | 0.088 | 0.231 | <0.001 | <0.001 | ns |
| 49:2 | 0.030 | 0.019 | 0.080 | 0.202 | 0.086 | 0.317 | 0.031 | 0.006 | 0.085 | 0.153 | 0.105 | 0.249 | <0.001 | <0.001 | ns |
| 49:3 | 0.005 | 0.002 | 0.019 | 0.056 | 0.033 | 0.099 | 0.004 | 0.003 | 0.010 | 0.046 | 0.032 | 0.070 | <0.001 | <0.001 | ns |
| 50:1 | 3.090 | 1.585 | 9.281 | 13.162 | 5.734 | 25.138 | 3.950 | 0.385 | 7.828 | 12.768 | 6.628 | 18.675 | <0.001 | 0.001 | ns |
| 50:2 | 4.306 | 2.504 | 13.06 | 20.977 | 8.418 | 33.196 | 4.990 | 0.856 | 11.172 | 18.376 | 10.430 | 28.089 | <0.001 | <0.001 | ns |
| 50:3 | 1.713 | 1.138 | 4.634 | 8.033 | 3.744 | 11.998 | 1.823 | 0.725 | 4.122 | 6.389 | 4.134 | 10.297 | <0.001 | <0.001 | ns |
| 50:4 | 0.306 | 0.187 | 0.669 | 1.555 | 0.964 | 2.559 | 0.333 | 0.256 | 0.628 | 1.359 | 0.890 | 2.092 | <0.001 | <0.001 | ns |
| 50:5 | 0.050 | 0.027 | 0.082 | 0.438 | 0.285 | 0.733 | 0.061 | 0.031 | 0.084 | 0.362 | 0.204 | 0.530 | <0.001 | <0.001 | ns |
| 50:6 | 0.004 | 0.002 | 0.011 | 0.134 | 0.087 | 0.216 | 0.006 | 0.001 | 0.024 | 0.113 | 0.060 | 0.193 | <0.001 | <0.001 | ns |
| 51:1 | 0.030 | 0.017 | 0.083 | 0.188 | 0.079 | 0.317 | 0.039 | 0.000 | 0.084 | 0.139 | 0.079 | 0.217 | <0.001 | <0.001 | ns |
| 51:2 | 0.156 | 0.086 | 0.414 | 0.616 | 0.256 | 1.086 | 0.178 | 0.030 | 0.435 | 0.521 | 0.291 | 0.887 | <0.001 | <0.001 | ns |
| 51:3 | 0.101 | 0.075 | 0.244 | 0.343 | 0.166 | 0.558 | 0.119 | 0.049 | 0.257 | 0.268 | 0.186 | 0.479 | <0.001 | 0.001 | ns |
| 51:4 | 0.033 | 0.029 | 0.070 | 0.115 | 0.063 | 0.169 | 0.045 | 0.031 | 0.069 | 0.088 | 0.064 | 0.138 | <0.001 | <0.001 | ns |
| 52:2 | 16.682 | 8.122 | 48.49 | 63.034 | 22.77 | 100.34 | 21.01 | 2.565 | 50.211 | 58.321 | 27.019 | 86.377 | 0.001 | 0.005 | ns |
| 52:3 | 15.438 | 10.60 | 37.40 | 44.217 | 19.64 | 69.597 | 18.32 | 6.226 | 37.384 | 37.125 | 22.402 | 58.764 | <0.001 | 0.010 | ns |
| 52:4 | 5.430 | 3.786 | 11.45 | 13.572 | 6.750 | 20.062 | 7.060 | 4.996 | 11.027 | 10.175 | 7.450 | 17.246 | <0.001 | 0.019 | ns |
| 52:5 | 0.955 | 0.590 | 1.607 | 2.604 | 1.496 | 4.149 | 1.163 | 0.700 | 1.578 | 1.983 | 1.391 | 3.296 | <0.001 | 0.002 | ns |
|  |  |  |  |  |  |  |  |  |  |  |  |  |  |  |  |
|  |  |  |  |  |  |  |  |  |  |  |  |  |  |  |  |
|  | **Water** | | | **LMCD Water** | | | **DEN** | | | **LMCD DEN** | | | ***P***_Water_ | ***P***_DEN_ | ***P***_LMCD_ |
| TG | Median | Min | Max | Median | Min | Max | Median | Min | Max | Median | Min | Max |  |  |  |
| 52:6 | 0.105 | 0.072 | 0.194 | 0.602 | 0.354 | 0.927 | 0.153 | 0.077 | 0.251 | 0.460 | 0.290 | 0.661 | <0.001 | <0.001 | ns |
| 52:7 | 0.011 | 0.006 | 0.025 | 0.089 | 0.064 | 0.161 | 0.016 | 0.008 | 0.051 | 0.078 | 0.045 | 0.126 | <0.001 | <0.001 | ns |
| 53:2 | 0.114 | 0.065 | 0.294 | 0.460 | 0.190 | 0.814 | 0.144 | 0.022 | 0.332 | 0.386 | 0.218 | 0.592 | <0.001 | 0.003 | ns |
| 53:3 | 0.148 | 0.098 | 0.336 | 0.483 | 0.201 | 0.779 | 0.184 | 0.051 | 0.392 | 0.392 | 0.223 | 0.620 | <0.001 | 0.006 | ns |
| 53:4 | 0.082 | 0.061 | 0.168 | 0.259 | 0.122 | 0.407 | 0.105 | 0.055 | 0.197 | 0.203 | 0.141 | 0.352 | <0.001 | 0.003 | ns |
| 53:5 | 0.067 | 0.055 | 0.144 | 0.248 | 0.128 | 0.374 | 0.097 | 0.042 | 0.144 | 0.210 | 0.146 | 0.316 | <0.001 | <0.001 | ns |
| 54:3 | 4.980 | 2.857 | 10.98 | 17.223 | 7.369 | 31.050 | 6.594 | 1.267 | 15.135 | 18.395 | 7.954 | 27.378 | <0.001 | 0.004 | ns |
| 54:4 | 4.751 | 3.241 | 9.128 | 14.420 | 7.826 | 29.282 | 6.074 | 2.815 | 12.651 | 15.097 | 7.589 | 22.483 | <0.001 | 0.006 | ns |
| 54:5 | 2.646 | 1.660 | 4.449 | 10.877 | 6.221 | 20.835 | 3.209 | 2.252 | 5.188 | 8.788 | 5.683 | 14.651 | <0.001 | <0.001 | ns |
| 54:6 | 0.932 | 0.538 | 1.576 | 5.409 | 2.693 | 8.031 | 1.464 | 0.705 | 2.028 | 3.363 | 2.498 | 6.104 | <0.001 | <0.001 | 0.042 |
| 54:7 | 0.144 | 0.090 | 0.278 | 0.871 | 0.454 | 1.285 | 0.238 | 0.115 | 0.534 | 0.593 | 0.409 | 0.960 | <0.001 | <0.001 | ns |
| 55:2 | 0.011 | 0.004 | 0.039 | 0.062 | 0.036 | 0.148 | 0.014 | 0.000 | 0.033 | 0.060 | 0.031 | 0.099 | <0.001 | <0.001 | ns |
| 55:3 | 0.039 | 0.028 | 0.089 | 0.166 | 0.080 | 0.329 | 0.050 | 0.012 | 0.118 | 0.156 | 0.078 | 0.255 | <0.001 | 0.002 | ns |
| 55:4 | 0.033 | 0.026 | 0.072 | 0.157 | 0.089 | 0.348 | 0.043 | 0.017 | 0.080 | 0.147 | 0.080 | 0.243 | <0.001 | <0.001 | ns |
| 55:5 | 0.024 | 0.018 | 0.054 | 0.209 | 0.127 | 0.462 | 0.034 | 0.018 | 0.055 | 0.182 | 0.114 | 0.279 | <0.001 | <0.001 | ns |
| 56:3 | 0.496 | 0.287 | 0.988 | 2.235 | 0.875 | 4.356 | 0.549 | 0.079 | 1.162 | 2.153 | 0.899 | 3.235 | <0.001 | <0.001 | ns |
| 56:4 | 0.545 | 0.357 | 1.023 | 3.202 | 1.518 | 6.985 | 0.635 | 0.162 | 1.204 | 3.085 | 1.328 | 4.922 | <0.001 | <0.001 | ns |
| 56:5 | 0.523 | 0.371 | 1.167 | 6.741 | 3.988 | 15.269 | 0.730 | 0.376 | 1.256 | 6.164 | 3.274 | 9.574 | <0.001 | <0.001 | ns |
| 56:6 | 0.536 | 0.394 | 1.213 | 8.518 | 5.157 | 19.815 | 0.817 | 0.589 | 1.508 | 7.126 | 4.242 | 10.825 | <0.001 | <0.001 | ns |
| 56:7 | 0.494 | 0.283 | 1.042 | 5.306 | 2.966 | 9.714 | 0.960 | 0.495 | 1.557 | 3.625 | 2.527 | 5.890 | <0.001 | <0.001 | ns |
| 56:8 | 0.252 | 0.149 | 0.559 | 1.499 | 0.678 | 2.064 | 0.592 | 0.239 | 0.990 | 0.901 | 0.701 | 1.855 | <0.001 | 0.011 | 0.043 |
| 58:3 | 0.045 | 0.029 | 0.083 | 0.266 | 0.101 | 0.605 | 0.038 | 0.008 | 0.066 | 0.229 | 0.104 | 0.385 | <0.001 | <0.001 | ns |
| 58:5 | 0.040 | 0.024 | 0.077 | 0.970 | 0.450 | 2.176 | 0.039 | 0.022 | 0.079 | 0.768 | 0.324 | 1.472 | <0.001 | <0.001 | ns |
| 58:6 | 0.082 | 0.056 | 0.226 | 2.275 | 1.199 | 5.733 | 0.128 | 0.072 | 0.222 | 1.775 | 0.929 | 3.098 | <0.001 | <0.001 | ns |
| 58:7 | 0.129 | 0.083 | 0.265 | 2.503 | 1.389 | 6.420 | 0.184 | 0.141 | 0.360 | 2.012 | 1.135 | 3.208 | <0.001 | 0.001 | ns |
| 58:8 | 0.135 | 0.089 | 0.276 | 1.766 | 1.091 | 3.691 | 0.267 | 0.155 | 0.469 | 1.283 | 0.864 | 1.964 | <0.001 | <0.001 | ns |
| 60:8 | 0.016 | 0.009 | 0.046 | 0.565 | 0.250 | 1.452 | 0.029 | 0.020 | 0.058 | 0.431 | 0.213 | 0.700 | <0.001 | 0.001 | ns |
| 60:9 | 0.019 | 0.013 | 0.047 | 0.541 | 0.280 | 1.339 | 0.036 | 0.025 | 0.070 | 0.400 | 0.251 | 0.606 | <0.001 | 0.001 | ns |

Supplementary Table 3: Proportion of triacylglycerol (TG) species in % of total TG. Median value, minimum (Min) and maximum (Max) of % TG are given. The *P* values for comparison of water injected mice and DEN injected mice fed the control chow are listed. Comparison of water injected mice and DEN injected mice fed the LMCD diet did not identify significant differences. The arrow indicates whether the respective TG is higher or lower in the liver of the DEN injected mouse.

|  | Water | | | LMCD Water | | | DEN | | | LMCD DEN | | | ***P***_Control diet_ |
| --- | --- | --- | --- | --- | --- | --- | --- | --- | --- | --- | --- | --- | --- |
| % TG of total TG | Median | Min | Max | Median | Min | Max | Median | Min | Max | Median | Min | Max |  |
| 49:1 | 0.04 | 0.03 | 0.05 | 0.06 | 0.05 | 0.07 | 0.04 | 0.01 | 0.04 | 0.06 | 0.05 | 0.07 | 0.006 ↓ |
| 50:1 | 5.61 | 3.45 | 5.95 | 4.82 | 3.33 | 5.45 | 4.52 | 1.18 | 5.78 | 4.84 | 3.53 | 5.61 | 0.044 ↓ |
| 50:2 | 7.00 | 5.46 | 7.93 | 6.91 | 6.08 | 8.08 | 6.27 | 2.63 | 6.81 | 7.40 | 6.23 | 8.11 | 0.002 ↓ |
| 50:3 | 2.68 | 2.35 | 2.81 | 2.88 | 2.43 | 3.65 | 2.30 | 2.06 | 2.61 | 2.86 | 2.29 | 3.47 | 0.015 ↓ |
| 56:8 | 0.34 | 0.16 | 0.82 | 0.48 | 0.39 | 1.29 | 0.63 | 0.27 | 2.72 | 0.50 | 0.27 | 1.00 | 0.046 ↑ |
| 58:8 | 0.17 | 0.11 | 0.29 | 0.74 | 0.42 | 0.96 | 0.31 | 0.18 | 0.95 | 0.61 | 0.45 | 0.97 | 0.033 ↑ |

Supplementary Table 4: Levels of free cholesterol (FC), cholesteryl ester (CE) and total cholesterol are given in nmol/mg. Median value, minimum (Min) and maximum (Max) are listed. The *P* values for comparison of water injected mice (*P*_Water_) and DEN injected mice (*P*_DEN_) fed the control chow or LMCD diet are listed. Comparison of the mice groups fed the control diet (P_Control_ _diet_) or LMCD diet (P_LMCD_) is also given. Not significant, ns.

|  | **Water** | | | **LMCD Water** | | | **DEN** | | | **LMCD DEN** | | | ***P***_Water_ | ***P***_DEN_ | ***P***_Control diet_ | ***P***_LMCD_ |
| --- | --- | --- | --- | --- | --- | --- | --- | --- | --- | --- | --- | --- | --- | --- | --- | --- |
|  | Median | Min | Max | Median | Min | Max | Median | Min | Max | Median | Min | Max |  |  |  |  |
| FC | 4.31 | 2.65 | 5.27 | 4.79 | 3.64 | 5.79 | 4.44 | 3.42 | 5.54 | 4.24 | 3.18 | 5.80 | ns | ns | ns | ns |
| CE | 4.34 | 1.80 | 6.85 | 2.72 | 0.93 | 3.74 | 3.64 | 1.50 | 8.37 | 2.82 | 1.47 | 5.24 | 0.029 | ns | ns | ns |
| Cholesterol | 8.66 | 5.68 | 12.12 | 7.05 | 6.10 | 8.55 | 8.08 | 5.29 | 13.10 | 7.10 | 5.08 | 9.52 | ns | ns | ns | ns |

Supplementary Table 5: Levels of diacylglycerol (DG), phosphatidylcholine (PC), phosphatidylethanolamine (PE), lysophosphatidylcholine (LPC), phosphatidylserine (PS) and phosphatidylinositol (PI) are given in nmol/mg. Median value, minimum (Min) and maximum (Max) are listed. The *P* values for comparison of water injected mice (*P*_Water_) and DEN injected mice (*P*_DEN_) fed the control chow or LMCD diet are listed. *P* values for the comparison of the mice groups fed the control diet (P_Control_ _diet_) is also given. Comparison of water injected mice and DEN injected mice fed the LMCD diet did not identify significant differences. Not significant, ns; Sat, saturated; MU, monounsaturated; PU, polyunsaturated.

|  | **Water** | | | **LMCD Water** | | | **DEN** | | | **LMCD DEN** | | | ***P***_Water_ | ***P***_DEN_ | ***P***_Control diet_ |
| --- | --- | --- | --- | --- | --- | --- | --- | --- | --- | --- | --- | --- | --- | --- | --- |
| Lipid class nmol/mg | Median | Min | Max | Median | Min | Max | Median | Min | Max | Median | Min | Max |  |  |  |
| Sat DG | 0.062 | 0.029 | 0.125 | 0.181 | 0.074 | 0.285 | 0.052 | 0.018 | 0.097 | 0.161 | 0.081 | 0.253 | <0.001 | <0.001 | ns |
| MU DG | 1.122 | 0.486 | 1.989 | 2.058 | 0.904 | 3.491 | 0.795 | 0.183 | 1.624 | 2.193 | 0.919 | 3.047 | 0.010 | 0.001 | ns |
| PU DG | 4.220 | 1.621 | 6.831 | 4.888 | 3.450 | 9.185 | 2.036 | 1.428 | 5.172 | 4.781 | 3.203 | 6.531 | ns | 0.013 | ns |
| DG | 5.879 | 2.136 | 8.236 | 7.290 | 4.938 | 12.96 | 2.886 | 1.706 | 6.893 | 7.134 | 4.203 | 9.567 | ns | 0.003 | ns |
| Sat PC | 0.222 | 0.144 | 0.283 | 0.190 | 0.141 | 0.222 | 0.206 | 0.151 | 0.250 | 0.168 | 0.117 | 0.253 | ns | ns | ns |
| MU PC | 1.179 | 0.717 | 1.607 | 1.392 | 1.046 | 1.697 | 0.904 | 0.800 | 1.410 | 1.560 | 1.245 | 2.175 | ns | <0.001 | ns |
| PU PC | 15.16 | 11.08 | 21.11 | 12.22 | 7.584 | 13.15 | 13.64 | 8.966 | 18.74 | 10.592 | 6.075 | 16.28 | 0.004 | ns | ns |
| PC | 16.54 | 11.94 | 23.10 | 14.03 | 8.824 | 14.91 | 14.77 | 12.80 | 20.40 | 12.473 | 10.69 | 18.60 | 0.005 | ns | ns |
| MU PE | 0.050 | 0.028 | 0.070 | 0.062 | 0.046 | 0.083 | 0.033 | 0.022 | 0.066 | 0.072 | 0.038 | 0.101 | ns | <0.001 | ns |
| PU PE | 6.183 | 4.486 | 8.377 | 6.145 | 4.068 | 6.825 | 6.138 | 3.888 | 7.630 | 5.431 | 3.682 | 8.129 | ns | ns | ns |
| PE | 6.233 | 4.524 | 8.447 | 6.215 | 4.129 | 6.885 | 6.168 | 3.915 | 7.696 | 5.510 | 3.754 | 8.193 |  |  | ns |
| Sat LPC | 0.390 | 0.200 | 0.479 | 0.287 | 0.227 | 0.337 | 0.292 | 0.192 | 0.448 | 0.241 | 0.169 | 0.393 | 0.032 | ns | ns |
| MU LPC | 0.084 | 0.037 | 0.104 | 0.065 | 0.055 | 0.088 | 0.046 | 0.032 | 0.111 | 0.063 | 0.047 | 0.104 | ns | ns | 0.046 |
| PU LPC | 0.344 | 0.176 | 0.483 | 0.246 | 0.165 | 0.295 | 0.213 | 0.144 | 0.477 | 0.200 | 0.148 | 0.350 | 0.014 | ns | ns |
| LPC | 0.835 | 0.413 | 1.066 | 0.594 | 0.447 | 0.710 | 0.561 | 0.388 | 1.036 | 0.498 | 0.366 | 0.827 | 0.024 | ns | ns |
| MU PS | 0.032 | 0.023 | 0.046 | 0.044 | 0.028 | 0.055 | 0.031 | 0.021 | 0.043 | 0.040 | 0.027 | 0.066 | ns | 0.014 | ns |
| PU PS | 2.388 | 1.744 | 3.113 | 2.110 | 1.592 | 2.363 | 2.304 | 1.600 | 2.867 | 1.987 | 1.516 | 2.902 | ns | ns | ns |
| PS | 2.423 | 1.769 | 3.152 | 2.145 | 1.642 | 2.406 | 2.335 | 1.620 | 2.906 | 2.028 | 1.561 | 2.968 | ns | ns | ns |
| MU PI | 0.015 | 0.010 | 0.021 | 0.021 | 0.013 | 0.032 | 0.013 | 0.008 | 0.017 | 0.023 | 0.015 | 0.034 | 0.011 | <0.001 | ns |
| PU PI | 5.873 | 3.719 | 7.035 | 5.027 | 3.694 | 6.368 | 5.535 | 3.804 | 7.425 | 4.978 | 3.226 | 7.629 | ns | ns | ns |
| PI | 5.897 | 3.732 | 7.053 | 5.044 | 3.725 | 6.393 | 5.548 | 3.816 | 7.449 | 5.002 | 3.257 | 7.668 | ns | ns | ns |

Supplementary Table 6: Lipidome of normal liver and tumor tissues of two mice (1, 2). Levels of free cholesterol (FC), cholesteryl ester (CE), phosphatidylcholine (PC), phosphatidylethanolamine (PE), lysophosphatidylcholine (LPC), phosphatidylserine (PS) and phosphatidylinositol (PI) are given in nmol/mg. Median value, minimum (Min) and maximum (Max) are listed. % change in the tumors is given in the final two columns. Phospholipid, PL.

|  | **Non-Tumor Tissue** | | | **Tumor Tissue 1** | | | **Tumor Tissue 2** | | | **Tumor Tissue 1/Non-Tumor Tissue x 100** | **Tumor Tissue 2/Non-Tumor Tissue x 100** |
| --- | --- | --- | --- | --- | --- | --- | --- | --- | --- | --- | --- |
| Lipids | Median | Min | Max | Median | Min | Max | Median | Min | Max |  |  |
| FC | 4.237 | 3.184 | 5.803 | 3.863 | 3.538 | 4.188 | 3.825 | 3.420 | 4.584 | 91 | 90 |
| CE | 2.824 | 1.468 | 5.238 | 1.580 | 1.574 | 1.585 | 1.866 | 1.630 | 3.857 | 56 | 66 |
| Sat PC | 0.168 | 0.117 | 0.253 | 0.237 | 0.176 | 0.297 | 0.116 | 0.111 | 0.221 | 141 | 69 |
| MU PC | 1.560 | 1.245 | 2.175 | 1.634 | 1.261 | 2.008 | 1.416 | 1.339 | 2.574 | 105 | 91 |
| PU PC | 10.592 | 6.075 | 16.277 | 11.073 | 8.211 | 13.934 | 7.348 | 6.241 | 9.103 | 105 | 69 |
| Total PC | 12.473 | 10.689 | 18.602 | 12.943 | 9.648 | 16.239 | 8.804 | 7.768 | 11.899 | 104 | 71 |
| Sat PE |  |  |  |  |  |  |  |  |  |  |  |
| MU PE | 0.072 | 0.038 | 0.101 | 0.227 | 0.195 | 0.259 | 0.168 | 0.163 | 0.219 | 317 | 234 |
| PU PE | 5.431 | 3.682 | 8.129 | 7.056 | 5.329 | 8.782 | 4.712 | 3.771 | 5.743 | 130 | 87 |
| Total PE | 5.510 | 3.754 | 8.193 | 7.283 | 5.524 | 9.041 | 4.880 | 3.934 | 5.962 | 132 | 89 |
| Sat LPC | 0.241 | 0.169 | 0.393 | 0.277 | 0.211 | 0.342 | 0.165 | 0.162 | 0.243 | 115 | 68 |
| MU LPC | 0.063 | 0.047 | 0.104 | 0.054 | 0.039 | 0.069 | 0.044 | 0.043 | 0.052 | 86 | 69 |
| PU LPC | 0.200 | 0.148 | 0.350 | 0.159 | 0.115 | 0.203 | 0.107 | 0.102 | 0.115 | 80 | 53 |
| Total LPC | 0.498 | 0.366 | 0.827 | 0.490 | 0.366 | 0.614 | 0.324 | 0.307 | 0.402 | 98 | 65 |
| MU PS | 0.040 | 0.027 | 0.066 | 0.041 | 0.030 | 0.052 | 0.034 | 0.033 | 0.042 | 102 | 84 |
| PU PS | 1.987 | 1.516 | 2.902 | 2.388 | 1.937 | 2.839 | 1.895 | 1.527 | 2.066 | 120 | 95 |
| Total PS | 2.028 | 1.561 | 2.968 | 2.429 | 1.967 | 2.891 | 1.928 | 1.561 | 2.108 | 120 | 95 |
| MU PI | 0.023 | 0.015 | 0.034 | 0.092 | 0.080 | 0.104 | 0.070 | 0.052 | 0.105 | 403 | 306 |
| PU PI | 4.978 | 3.226 | 7.629 | 6.070 | 5.112 | 7.029 | 4.193 | 3.576 | 4.821 | 122 | 84 |
| Total PI | 5.002 | 3.257 | 7.668 | 6.166 | 5.194 | 7.137 | 4.248 | 3.649 | 4.931 | 123 | 85 |
